# Description of *Sandythoa* gen. nov., a fish parasitic branchial cymothoid (Crustacea: Isopoda: Cymothoidae) from the Indian Ocean, with five species including one new species

**DOI:** 10.1007/s11230-024-10163-2

**Published:** 2024-07-07

**Authors:** Panakkool Thamban Aneesh, Niel Bruce, Ameri Kottarathil Helna, Appukuttannair Biju Kumar

**Affiliations:** 1 Blue Innovation Division, Seto Inland Sea Carbon Neutral Research Center, Graduate School of Integrated Sciences for Life, Hiroshima University, 5-8-1 Minato-machi, Takehara, Hiroshima, 725-0024 Japan; 2 Travancore Nature History Society (TNHS), MBRRA, Mathrubhumi Road, Vanchiyoor, Thiruvananthapuram, Kerala 695035 India; 3 Biodiversity and Geosciences Program, Queensland Museum, South Brisbane BC, PO Box: 3300, Brisbane, QLD 4101 Australia; 4 Water Research Group, Unit for Environmental Sciences and Management, North-West University, Private Bag X6001, Potchefstroom, 2520 South Africa; 5 Regional Forensic Science Laboratory, Kannur, Kerala 670002 India; 6 Department of Aquatic Biology and Fisheries, University of Kerala, Karyavattom, Thiruvananthapuram, Kerala 695 581 India

## Abstract

The comparative analysis of records of *Elthusa samariscii* (Shiino, 1951) from Japan and India, alongside corresponding illustrations, indicates that the records of *E. samariscii* from *Samaris cristatus* Gray in India represent a distinct and previously undescribed species. This study introduces *Sandythoa tiranga* **gen.** and **sp. nov.,** providing comprehensive descriptions of various lifecycle stages, including the female, male, transitional, premanca, and manca larvae. The following combinations of characters identify the genus: cephalon anterior margin with acute rostrum; pleonite 1 is distinctly narrow, not extending laterally; presence of a narrow gap between pleonites; antenna with more than 10 articles; maxilliped with oostegital lobe. *Sandythoa tiranga* **sp. nov.** is specifically identified along the southwest coast of India. Furthermore, we propose transferring the following species from *Elthusa*: *Sandythoa arnoglossi* (Trilles and Justine 2006) **comb. nov.;** *Sandythoa parabothi* (Trilles and Justine, 2004) **comb. nov.**; *Sandythoa samariscii* (Shiino, 1951) **comb. nov.**; *Sandythoa moritakii* (Saito and Yamauchi, 2016) **comb. nov.** A revised key to the global marine branchial cymothoid genera is provided.

## Introduction

The branchial fish parasitic isopod genus *Elthusa* Schioedte & Meinert, 1884, was recently revised by Aneesh et al. (2020a), and a restricted generic diagnosis was provided based on the type species, *Elthusa emarginata* (Bleeker, 1857). Thirteen species of *Elthusa* that did not fully conform to the new diagnosis were placed into *Elthusa incertae sedis,* while the remaining 26 species were retained in combination with *Elthusa* (See Aneesh et al., 2020a). That recent revision of *Elthusa* has allowed for a better understanding of the characters within the genus and for the allocation of species, firstly to the recently described *Glyptothoa* Helna, Aneesh, Kumar, & Ohtsuka, 2023 (three species), and here four species placed into a new genus (see Helna et al., 2023). Aneesh et al. (2023a, b) recently described *Elthusa aquabio* Aneesh, Helna, Raj, & Kumar, 2023 and *Elthusa nemo* Aneesh, Helna, Raj, & Kumar, 2023 from the southwest coast of India.

*Livoneca samariscii* Shiino, 1951 was originally described from the samarid fish *Samariscus japonicus* Kamohara from Kochi, Japan. Bruce (1990) transferred it into the genus *Elthusa*. *Elthusa samariscii* (Shiino, 1951) was subsequently reported and redescribed from Kerala, southwest coast of India, by Kumar and Bruce (1997) and Aneesh et al. (2020a) from another species of samarid, *Samaris cristatus* Gray. Aneesh et al. (2020a), retained the species within *Elthusa*, but as *incertae sedis*.

The present study initially set out to place *Elthusa samariscii incertae sedis* from India into the correct genus by examining the type specimen of *E. samariscii* deposited by Shiino (1951). As the type material appears to be missing, we compared the Indian specimen to the description and illustrations of Shiino (1951) (see Fig. 18). Based on that description (Shiino, 1951), it became clear that the specimens from India identified as *Elthusa samariscii* belongs to a different species than *Elthusa samariscii* from Japan. Furthermore, in the process of describing the new species from *Samariscus cristatus* from India, it became apparent that a new genus was needed for the new species as well as four other species currently placed *incertae sedis* in *Elthusa*. Together with recently described *Glyptothoa*, the new genus described here brings the total number of accepted cymothoid genera to 42 (Helna et al. 2023; Aneesh et al. 2024). A revised key to the global marine branchial cymothoid genera is also provided.

## Materials and methods

Fresh isopod specimens were collected from the branchial cavity of cockatoo righteye flounder, *Samaris cristatus* Gray (Samaridae), caught by commercial trawlers operating from Neendakara (08° 30.0′ N 76° 53.30′ E), Kollam district, Kerala state, and Muttom, Tamil Nadu state southwest coast of India. Methods for collection, preservation, dissection, mounting, and drawings of appendages follow Aneesh et al. (2019, 2020b, 2021a, b, 2022; 2024). One ovigerous female was designated as the holotype and one paratype was minimally dissected to conserve the specimens (the dissected appendages were kept in separate vials along with the specimen). The specimens were microphotographed using a multi-focusing dissection microscope Leica-M205A and image capturing software (Leica Application Suit). Drawings were digital-inked using Adobe Illustrator and a WACOM CTL-472/K0-c drawing pad. Sources for the fish taxonomy and host nomenclature were FishBase (Froese & Pauly, 2024) and Catalogue of Fishes (Fricke et al., 2024). Classification of the cymothoid follows Brandt & Poore (2003). The type specimens are deposited in the Western Ghat Field Research Centre of the Zoological Survey of India, Kozhikode (ZSI/WGRC) and and PTA’s & AKH’s personal collection in India (CAH).

**Abbreviations:** RS, robust seta/e; BL, body length; W, width; ZSI/WGRC, Western Ghat Field Research Centre of Zoological Survey of India, Kozhikode.

## Results

### Taxonomy

**Suborder Cymothoida Wägele, 1989**

**Superfamily Cymothooidea Leach, 1814**

**Family Cymothoidae Leach, 1814**

**Genus** ***Sandythoa*** **gen. nov.**

**urn:lsid:zoobank.org:act:98058CA3-7B41-4C64-BA8E-6EDC5BDF0721 **                                                                                                                                              Type species:* Sandythoa tiranga* **sp. nov**.; original designation.

***Diagnosis of female* (bold = diagnostic characters).** Body vaulted dorsally, widest at pereonite 3. Cephalon not deeply immersed in pereonite 1, **anterior margin with acute rostral point; rostrum narrowly rounded, not folded.** Pereonites 2–7 coxae visible in dorsal view, pereonites 7 posterolateral margins partially concealing pleonite 1 and 2. Pleon short 15% BL, **less than 0.70 times as wide as maximum pereon width. Pleonites laterally separated by moderate gaps. Pleonite 1 laterally strongly reduced.** Antennula separated by rostrum, slender, with 8 articles, shorter than antenna. Antenna with more than 10 articles. Buccal cone obscuring antennal bases. Pereopods dactyli relatively short, strongly curved. Brood pouch from coxae 2–5. Pleopods not visible in dorsal view. Pleopod peduncle lateral lobes absent. Uropods short, extending approximately halfway along pleotelson lateral margin.

***Additional characters:*** Mandible palp articles all slender. Maxilla mesial lobe distinct (not fused). Maxillula with 4 acuminate terminal RS. Maxilliped with oostegital lobes; mouthparts not covered by oostegites of pereopod 1. Pereopodal bases each without a prominent carina, without setae; articles not dilated or expanded.

**Species included:** *Sandythoa tiranga ***sp. nov. (**type species); *Sandythoa arnoglossi* (Trilles and Justine 2006) **comb. nov.,** *Sandythoa moritakii* (Saito and Yamauchi, 2016) **comb. nov.,** *Sandythoa parabothi* (Trilles and Justine, 2004) **comb. nov.,** and *Sandythoa samariscii* (Shiino, 1951) **comb. nov.**

**Etymology:** The genus is named in honour of the late Alexander James Bruce, known to all as ‘Sandy’ (the Scottish diminutive of his first name), in tribute to his memory and in recognition of his significant contribution to the taxonomy of decapod crustaceans. The gender is feminine.

### Remarks

*Sandythoa* **gen. nov.** can be distinguished from all other branchial cymothoid genera by the following combinations of female characters: cephalon anterior margin with acute rostrum, rostrum narrowly rounded, not folded; pleonite 1 is laterally strongly reduced; the presence of a moderate gap between pleonites; pleon short 15% BL, less than 0.70 times as wide as maximum pereon width; antenna with more than 10 articles; maxilliped with oostegital lobe; pleopod peduncle lateral lobes absent; uropods short, extending approximately halfway along pleotelson lateral margin.

**Key to the marine branchial cymothoid genera of the world**

**1.** Body strongly distorted, asymmetrical in shape….............................................................................................**2**

**—** Body not distorted, bilaterally symmetrical/weakly to moderately asymmetrical.............................................**5**

**2.** Cephalon with rostral point, coxae equal or unequal on both sides of body*………………………….…….…..…..***3**

**—** Cephalon without rostral point, coxae more or less equal on both sides of body……..………………….…....**4**

**3.** Pereonites 1–4 of hunched side laterally much expanded from pereopodal bases; brood pouch extensively bulged ventrally; pleotelson asymmetrical……………….………………***Agarna* Schioedte & Meinert, 1884**

**—** Pereonites 1–4 of hunched side not expanded from pereopodal bases; brood pouch ventrally not bulged; pleotelson symmetrical …………………………………………….………***Cterissa* Schioedte & Meinert, 1884**

**4.** Antennula with fewer than five articles; pleon compressed and hunched…………………………………

……………………………………………………………………….***Kuna* Bunkley-Williams & Williams, 1986**

**—** Antennula with more than five articles; pleon immersed in pereon…………………………………………… ………………………………………………………………..….***Ryukyua* Williams & Bunkley-Williams, 1994**

**5.** Cephalon with rostral point ……………………………………………………..……*…………………………..***6**

**—** Cephalon without rostral point …………………………………….….………………………..….………....**9**

**6.** Oostigite 1 bilobed; brood pouch ventrally bulged…………………………………………...……………….**7**

**—** Oostigite 1 not bilobed; brood pouch not ventrally bulged………………..………………………………….**8**

**7.** Pereonites 4–7, lateral margin constricted in hunched side; pleopods not much larger, not visible in dorsal view; all coxae dorsally visible; pleon 1.0–1.2 times as wide as widest pereomere……………………………… …………………………………………………………***Glyptothoa* Helna, Aneesh, Kumar & Ohtsuka, 2023**

**—** Pereonites 4–7, lateral margin not constricted; pleopods very large; visible in dorsal view; coxae of pereonite 6 and 7not visible in dorsal view; pleon 0.87 times as wide as widest pereomere………………………………… ……………………………………………………………..***Brucethoa* Aneesh, Hadfield, Smit & Kumar, 2020**

8. Body dorsum not or weakly vaulted; short gaps between pleonites; pleonite 1 laterally strongly reduced; pleon less than 0.7 times as wide as widest pereomere…………………………………………...***Sandythoa* gen. nov.**

**—**Body dorsum vaulted; no gaps between pleonites; pleonite 1 laterally not reduced; pleon narrow (<0.5 times as wide as pereon)………………………………………..……….…………… ***Ichthyoxenos* Herklots, 1870**

**9.** Pleopodal peduncle with lateral lobe, ramus with fleshy lobe/coupling hooks……………………….….….**10**

**—** Pleopods simple, without lateral, fleshy lobes or coupling hooks………………………………….....….…**13**

**10.** Cephalon posterior margin trilobed; pleon not immersed in pereon; pleonites 1–5 progressively narrower………………………………………………………………………………………………………….**11**

**—** Cephalon posterior margin not trilobed; pleon immersed in pereon; pleonites 1–5 not progressively narrower ………………………………………………………………………………………………………………….**12**

**11.** Cephalon strongly tri-lobed; pleonites 1–3 lateral margins bilobed; pleopodal peduncle with branchiae…………………………………………………………………………..……. ***Livoneca* Leach, 1818**

**—** Cephalon weakly tri-lobed; pleonites 1–3 lateral margins not bilobed; pleopodal peduncle without branchiae…………………………………………………….………………………..…. ***Norileca*** **Bruce**, **1990**

**12.** Pereopods without robust setae; pereonite 1 produced into lobe along one or both lateral margins of head; Coxae of pereonites 2 and 3 medially inflated, much larger than remaining coxae……………………………. ………………………………………………………………………………..***Joryma*** **Bowman & Tareen, 1983**

**—** Pereopods with many fine robust setae; pereonite 1 antero-laterally produced into conical lobes; coxal plates well developed and projecting…………………………………….……………….…….***Pseudirona* Pillai, 1964**

**13.** Small gap between pleonites; body bilaterally symmetrical; dorsum vaulted; all coxae small; cephalon not deeply immersed in pereonite1; rotationally twisted pleon with narrow pleonite 1……………………………… ………….…………………………………………………………….…….***Catoessa* Schioedte & Meinert, 1884**

**—** No gap between pleonites; Body weakly to moderately asymmetrical; dorsum not or weakly vaulted; posterior coxae often large; cephalon immersed in pereonite 1………………………………………….……**14**

**14.** Uropods short, not reaching posterior of pleotelson; pleon wide (greater than 0.75 maximum width of pereon); antennula shorter than antenna……………………………..……***Elthusa* Schioedte & Meinert, 1884**

**—** Uropods mostly longer, reaching or extending posterior to pleotelson; pleon variable (0.52–1.01, maximum width of pereon); antennula longer than antenna…………………..…….…***Mothocya* A. Costa *in*** **Hope**, **1851**

## *Sandythoa tiranga* sp. nov.

urn:lsid:zoobank.org:act:98058CA3-7B41-4C64-BA8E-6EDC5BDF0721

 (Figs. 1, 2, 3, 4, 5, 6, 7, 8, 9, 10, 11, 12, 13, 14)

**Figure 1:**
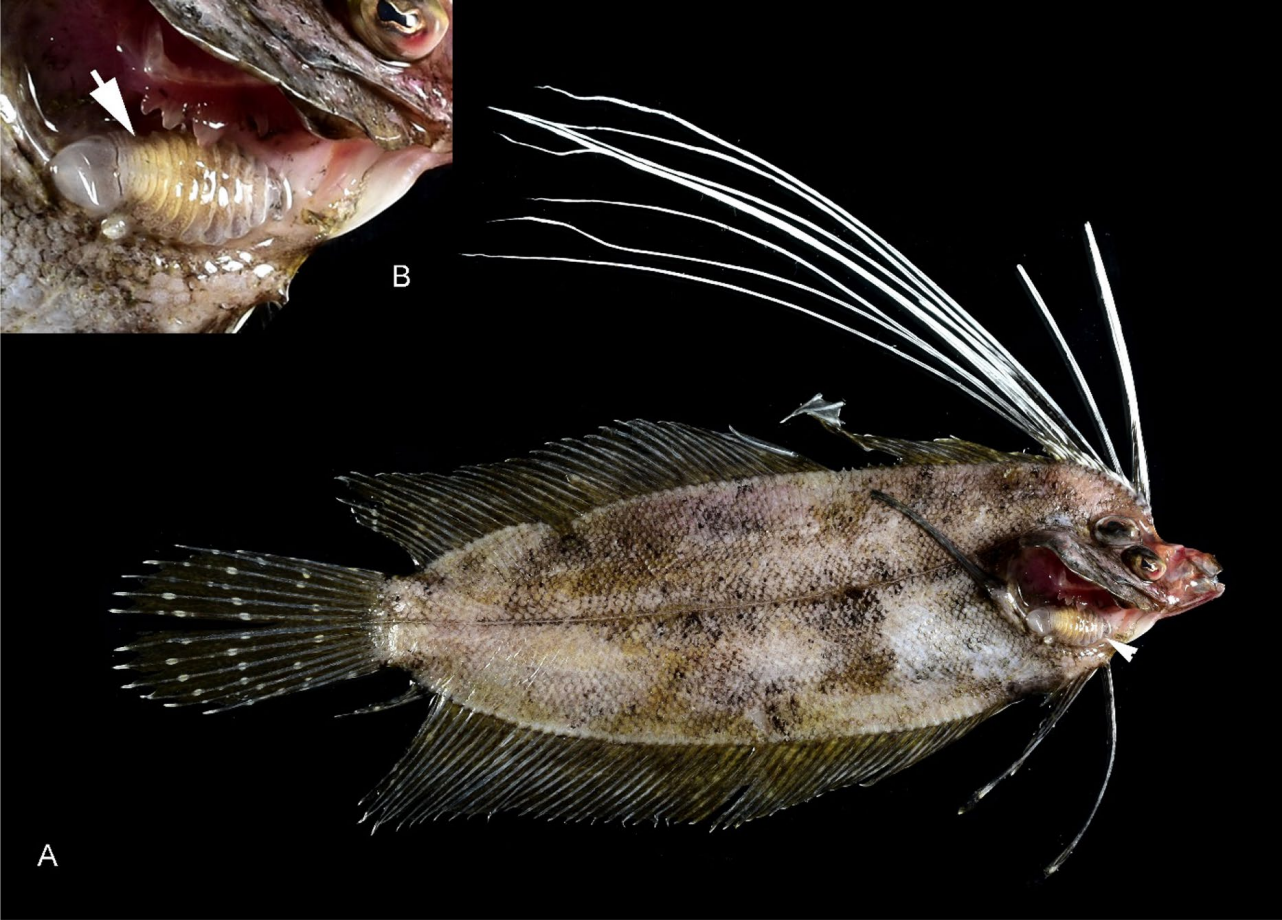
A–B, *Sandythoa tiranga* **gen.** and **sp. nov**., female in the branchial cavity of the host fish *Samaris cristatus* Gray (Samaridae).

**Figure 2:**
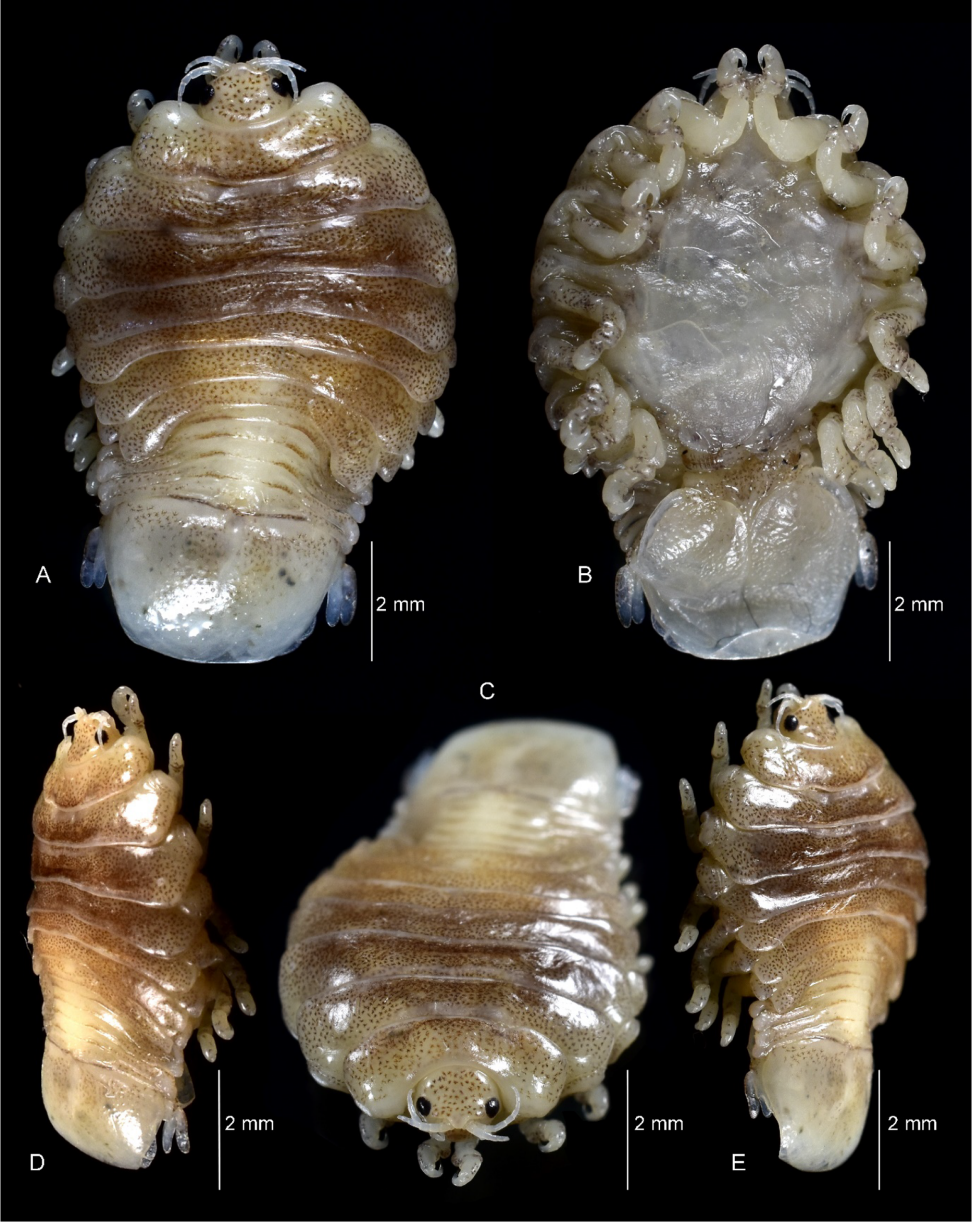
*Sandythoa tiranga* **gen.** and **sp. nov**., holotype female (Reg. No. ZSI/WGRC/IR. INV/26588). A, dorsal view; B, ventral view; C, dorso-frontal view; D–E, lateral views.

**Figure 3:**
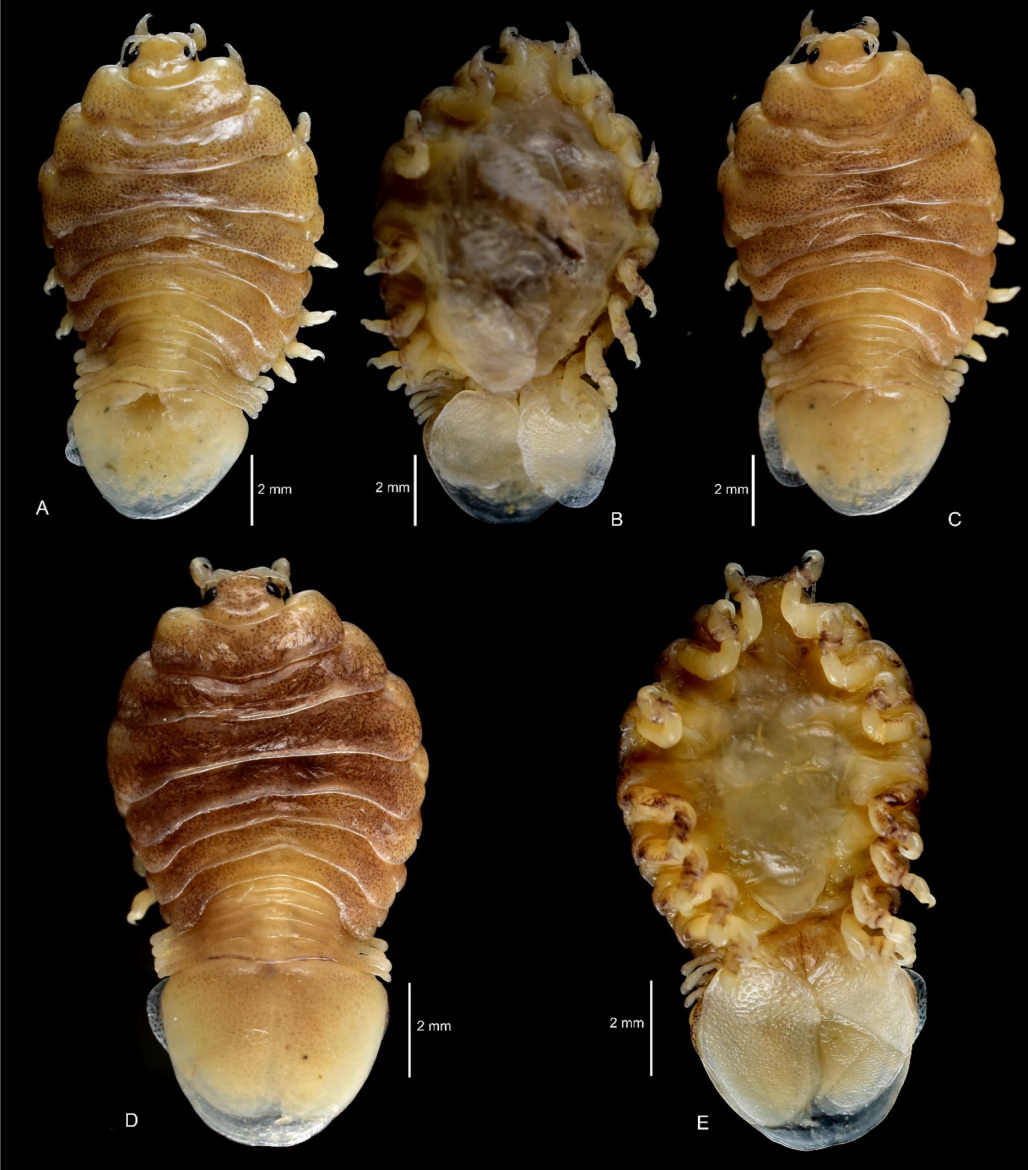
*Sandythoa tiranga* **gen.** and **sp. nov**., A–B, paratype female (Reg. No. CAH/INV/ISO 0319) dorsal and ventral view; C. paratype female (Reg. No. CAH/INV/ISO 0320) dorsal view; D–E, paratype female (Reg. No. CAH/INV/ISO 0321) dorsal and ventral view.

**Figure 4:**
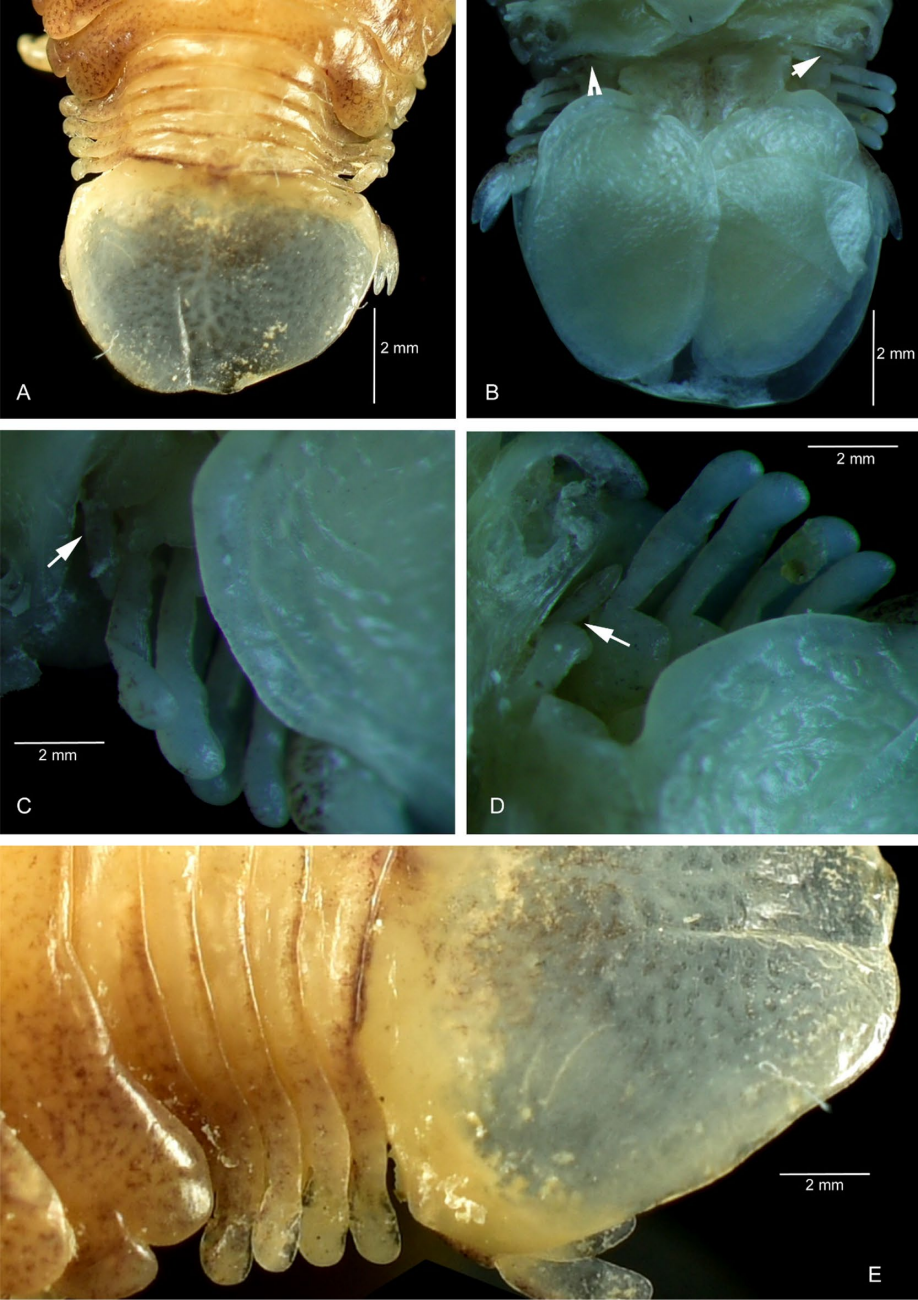
*Sandythoa tiranga* **gen.** and **sp. nov**., pleonites of ovigerous female showing the lateral gaps and highly reduced pleonite 1. A, dorsal view; B–D, ventral view showing reduced pleonite 1 (arrows); E, lateral view.

**Figure 5:**
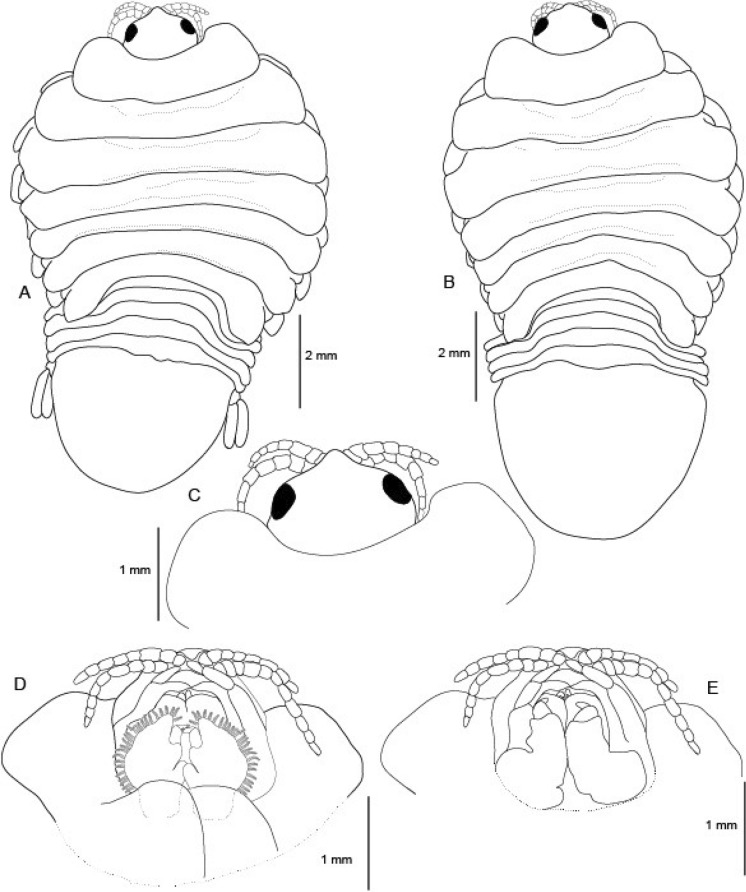
*Sandythoa tiranga* **gen.** and **sp. nov**., from *Samaris cristatus* (Samaridae). A, holotype female (Reg. No. ZSI/WGRC/IR.INV./26588); B, paratype female (CAH/INV/ISO 0322); (C–D), cephalon; C, dorsal view; D, ventral view of head of non-ovigerous female showing mouthparts; E, ventral view of head of ovigerous female showing mouthparts.

**Figure 6:**
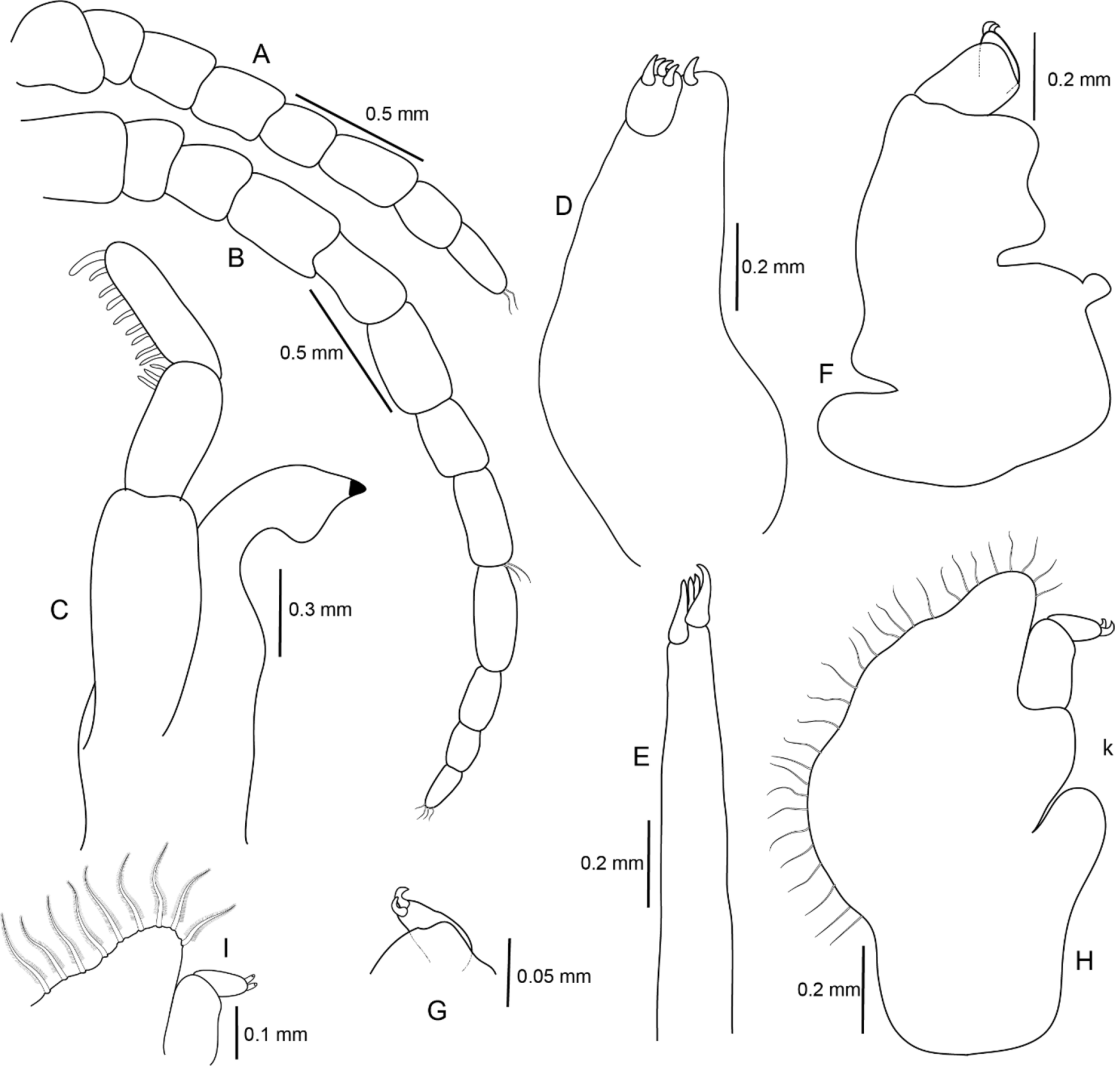
*Sandythoa tiranga* **gen.** and **sp. nov**., paratype female. A, antennae; B, antennule; C, mandible; D, maxilla; E, maxillule; F, maxilliped of non-ovigerous female; G, distal segment of maxilliped palp of non-ovigerous female; H, maxilliped of ovigerous female; I, distal segment of maxilliped palp and plumose setae of maxilliped of overigerous female.

**Figure 7:**
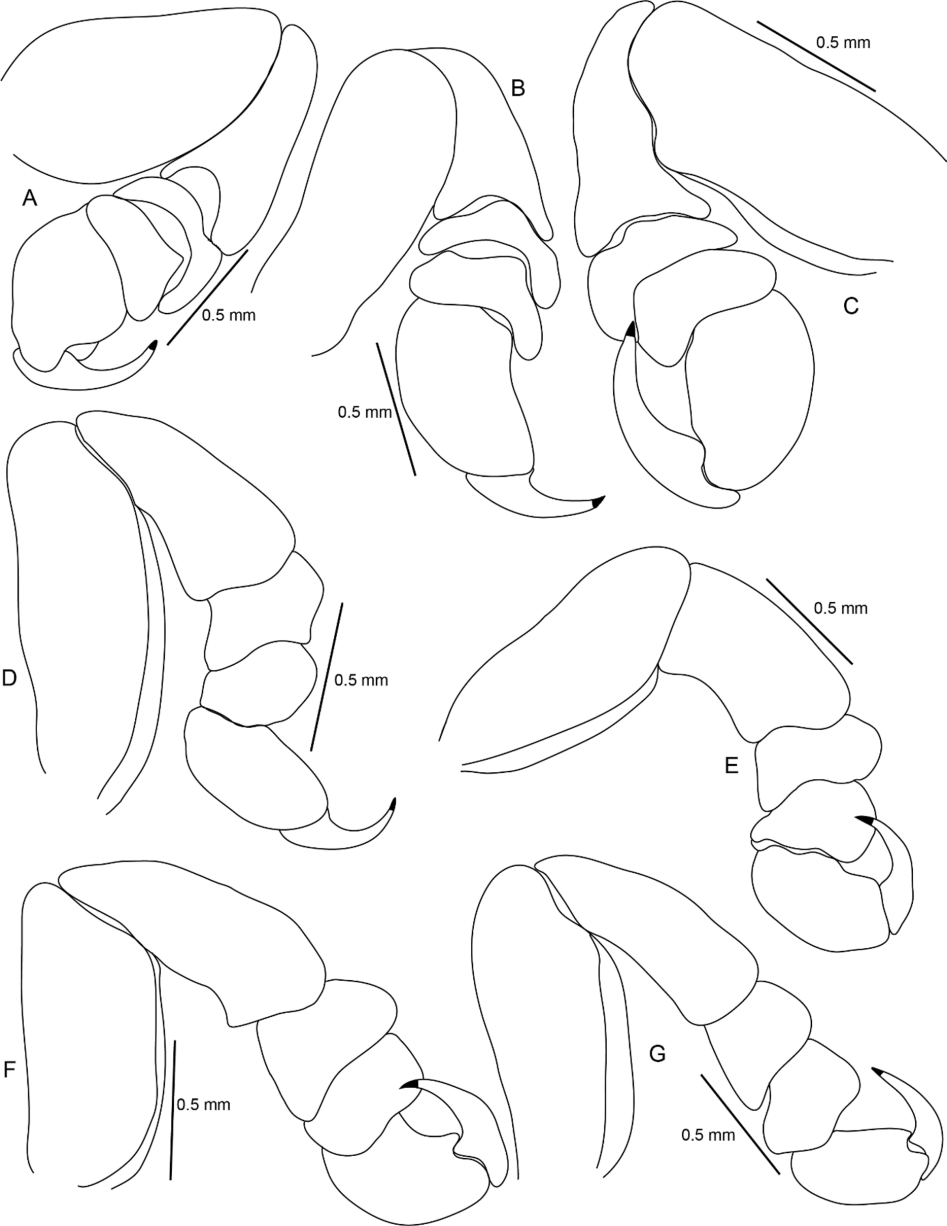
*Sandythoa tiranga* **gen.** and **sp. nov**., (A–G) pereopods 1–7.

**Figure 8:**
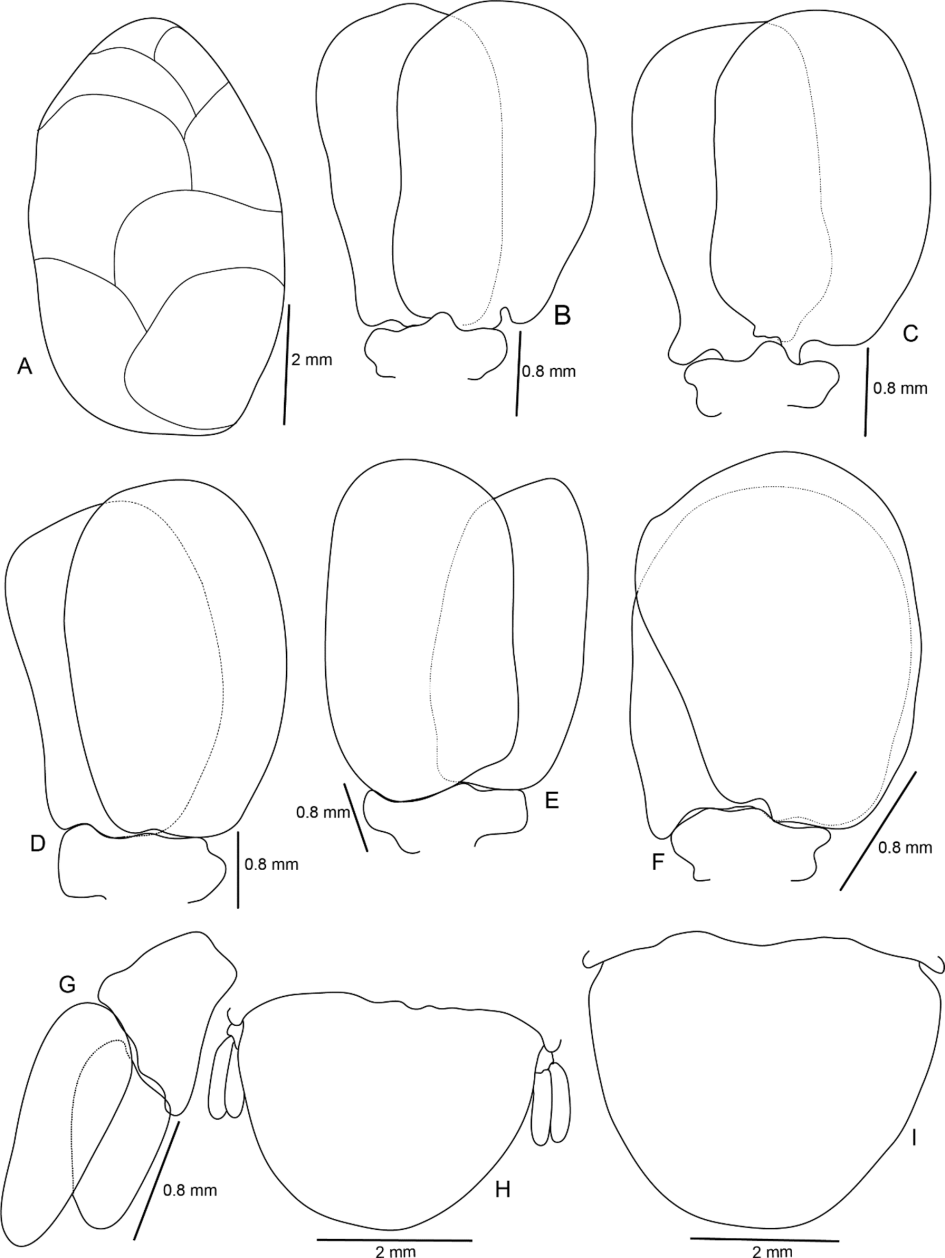
*Sandythoa tiranga* **gen.** and **sp. nov**., A, brood pouch; B–F, pleopods 1–5; G, uropod; H, pleotelson and uropods, I, pleotelson.

**Figure 9:**
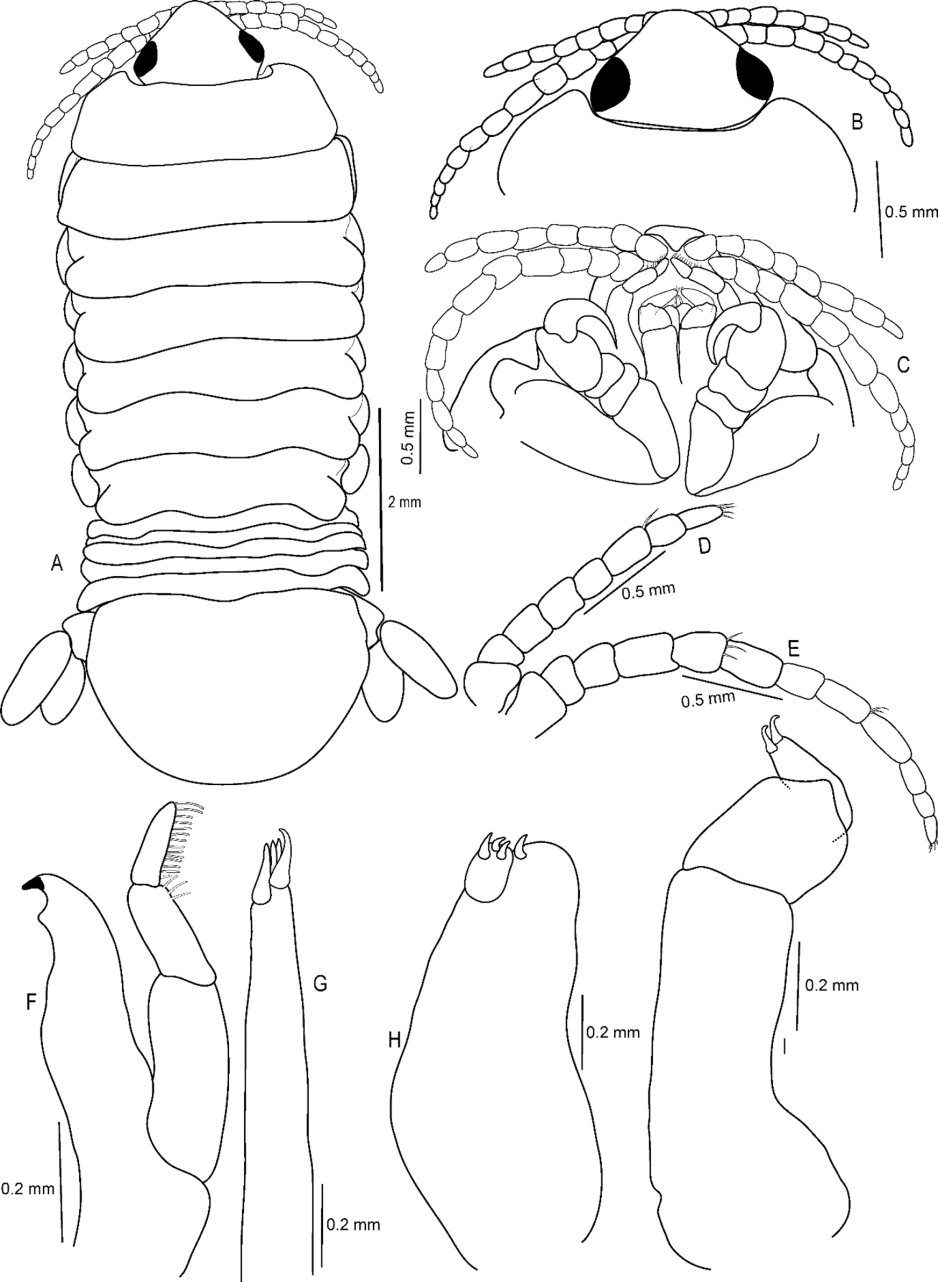
*Sandythoa tiranga* **gen.** and **sp. nov**., paratype male (Reg No ZSI/WGRC/IR/INV/11726), A, dorsal view; B–C, cephalon; B, dorsal view; C, ventral view; D, antennule; E, antenna; F, mandible; G, maxillule; H, maxilla; I, maxilliped.

**Figure 10:**
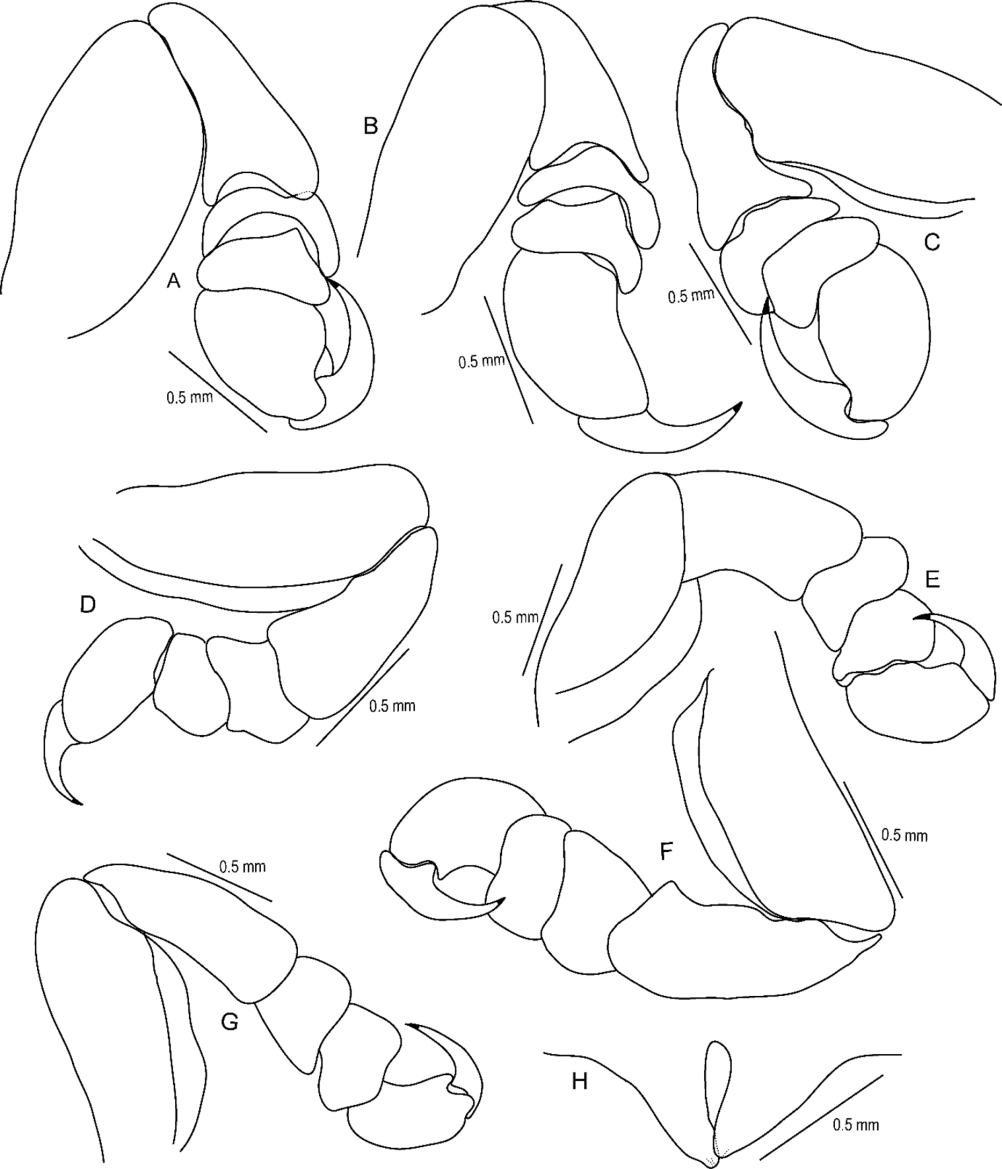
*Sandythoa tiranga* **gen.** and **sp. nov**., male (A–G), pereopods 1–7; H, penes.

**Figure 11:**
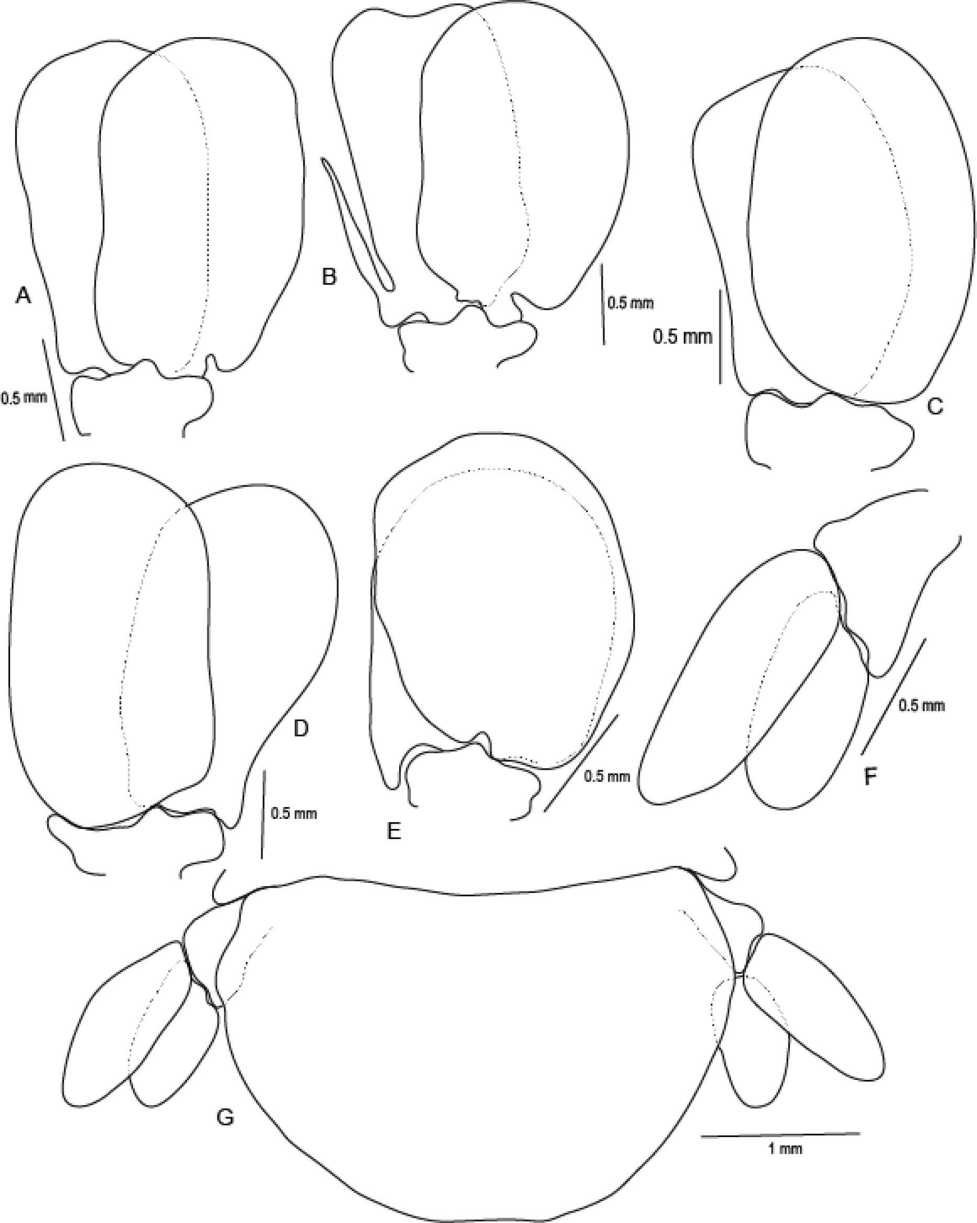
*Sandythoa tiranga* **gen.** and **sp. nov**., male. A–E, pleopods 1–5; F, uropod; G, pleotelson and uropods.

**Figure 12:**
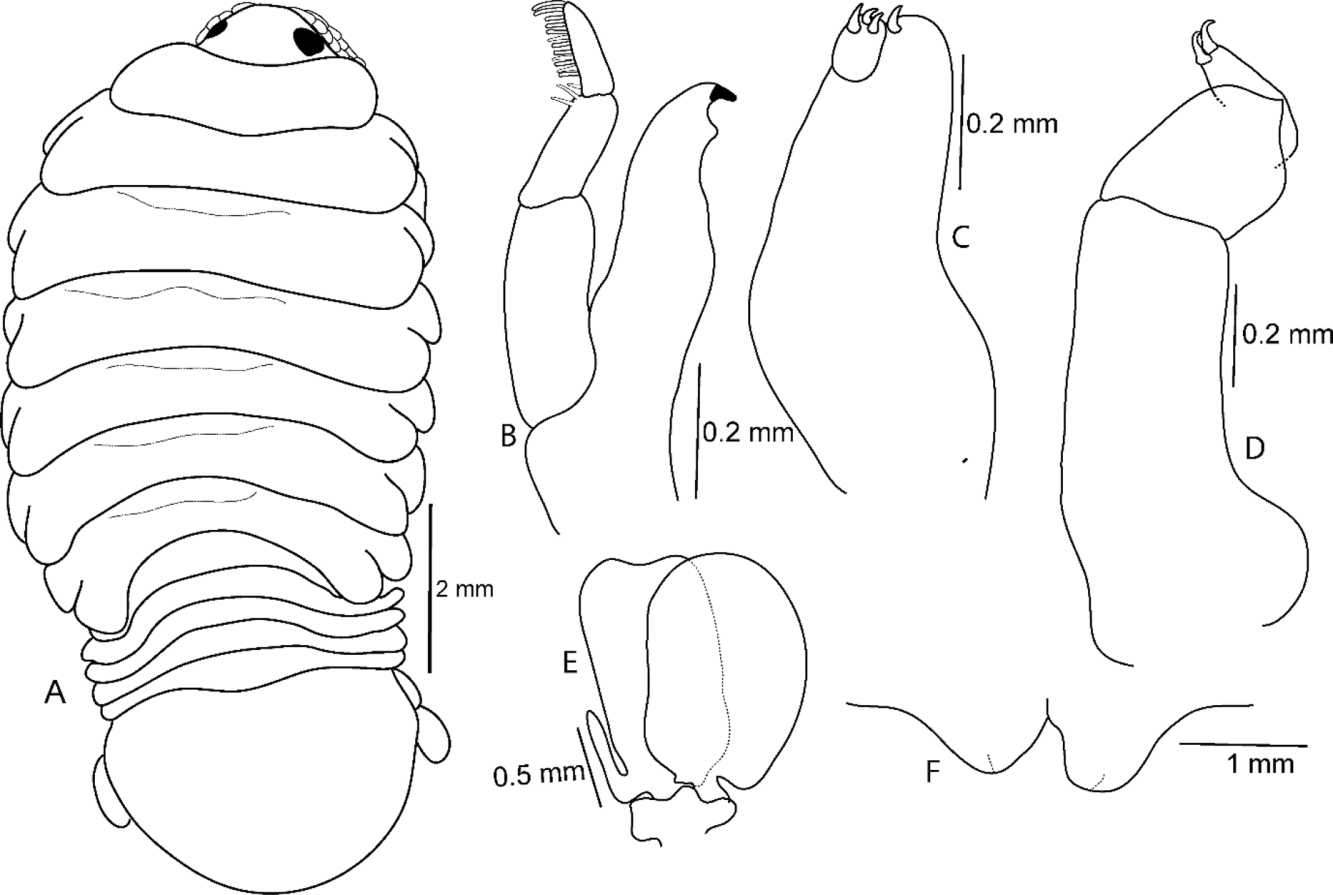
*Sandythoa tiranga* **gen.** and **sp. nov**., transitional stage. A, dorsal view; B, mandible; C, maxilla; D, maxilliped; E, pleopod 2; F, penes.

**Figure 13:**
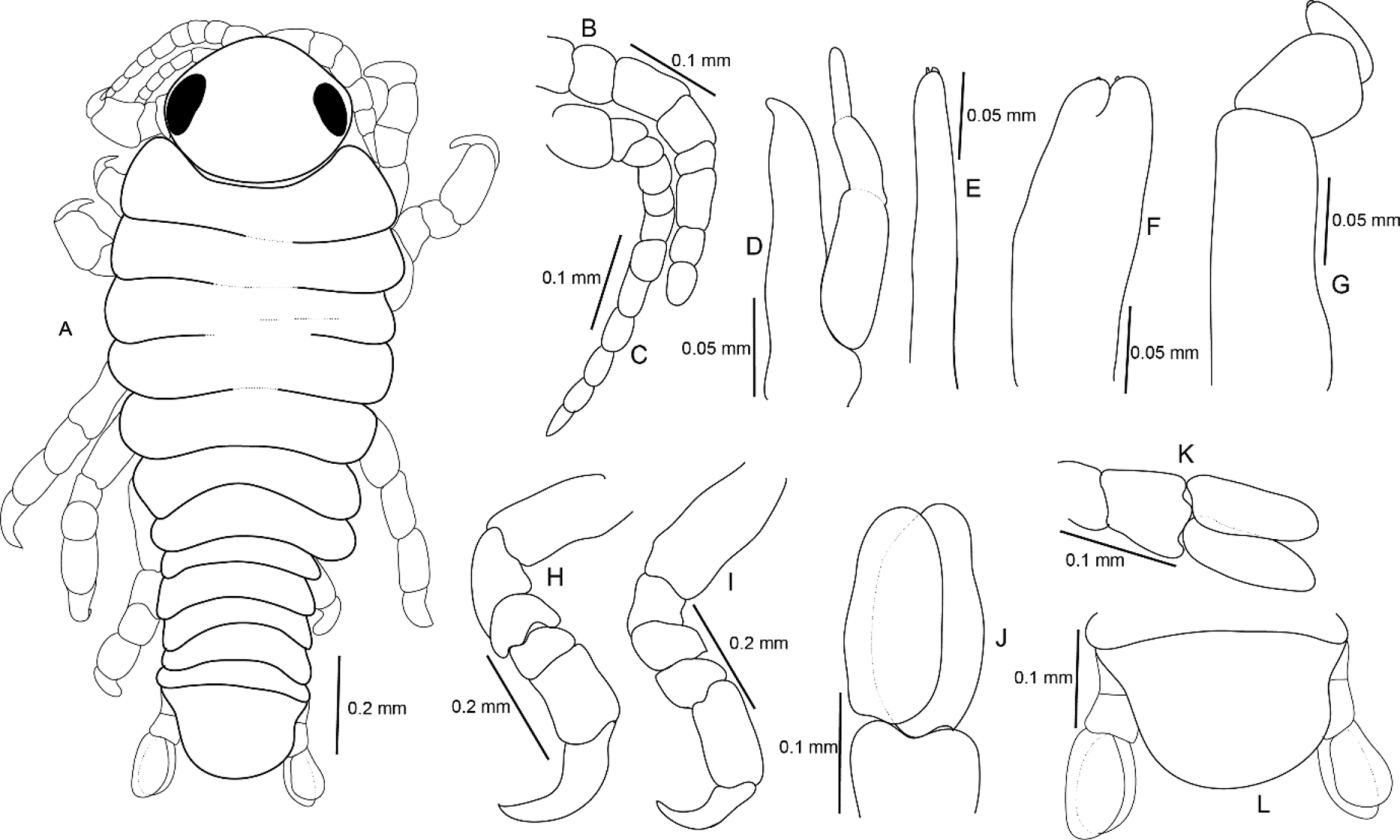
*Sandythoa tiranga* **gen.** and **sp. nov**., pre-manca larva. A, dorsal view; B antennule; C, antenna; D mandible; E maxillule; F maxilla; G maxilliped; H, pereopod 1; I, pereopod 6; J pleopod 1; K, uropod; L, pleotelson and uropods.

**Figure 14:**
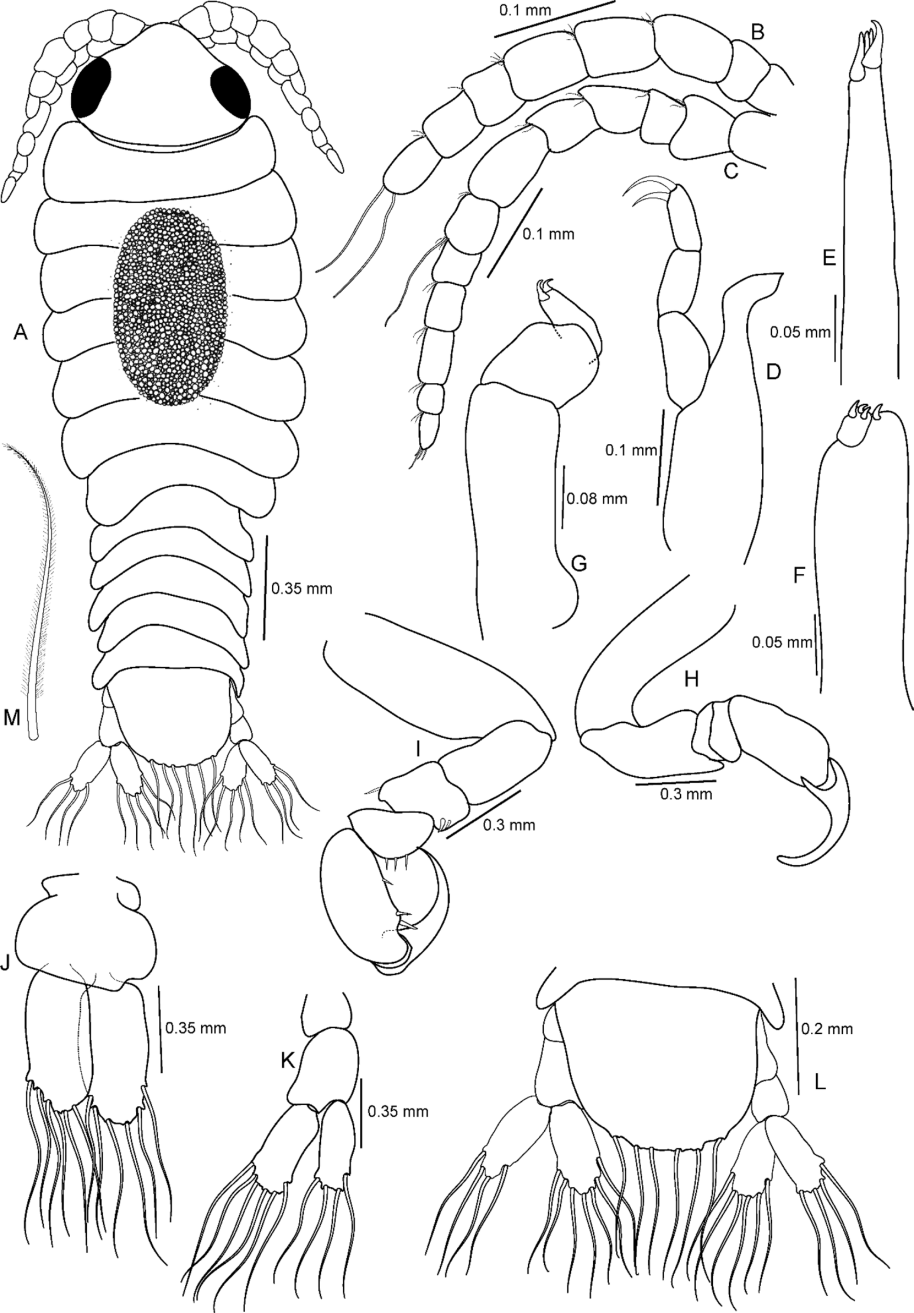
*Sandythoa tiranga* **gen.** and **sp. nov**., manca larva. A, dorsal view; B antennule; **C,** antenna; **D** mandible; **E** maxillule; **F** maxilla; **G** maxilliped; H, pereopod 1; I, pereopod 6; J pleopod 1; K, uropod; L, pleotelson and uropods.

*Elthusa samariscii*.— Kumar & Bruce, 1997: 780–787, figs 1–4.—Aneesh et al., 2020a, 2020b: 13–23, figs 11–22 [not *Elthusa samariscii* (Shiino, 1951)].

***Material examined:**** Holotype.* 1 ovigerous female (10.0 mm) from *Samaris cristatus* Gray (Samaridae), off Neendakara coast, 08°30.0’N, 76°53.30’E, Kerala, India, 29 December, 2019, coll. PT Aneesh and AK Helna (Reg. No. ZSI/WGRC/IR.INV./ 26588).

*Paratypes*: Same data as holotype with the following measurements and registration details: 1 female (ovigerous) (10.0 mm), from Muttom, southwest coast, India*,* 22 May 2018, (ZSI/WGRC/IR/INV/11723); 1 transitional stage (8.5 mm), from Muttom, southwest coast, India (8° 07′ 48.00″ N 77° 19′ 12.00″ E)*,* 17 July 2018, (ZSI/WGRC/IR/INV/11724); 1 ovigerous female (14.0 mm), from Muttom, southwest coast, India*,* 04 March 2018, (ZSI/WGRC/IR/INV/11725); 1 male (7.0 mm), from Neendakara fish landing center (08° 30.0′ N 76° 53.30′ E), Quilon, Kerala Coast, 17 August 2018, (ZSI/WGRC/IR/INV/11726); 1 male (8.0 mm), from Muttom, southwest coast, India*,* 04 March 2018, (ZSI/WGRC/IR/INV/11727); 1 ovigerous female (13.5.0 mm), from Muttom, southwest coast, India, 04 March 2018 (CAH/INV/ISO 0319); 1 ovigerous female (12.0 mm), from Muttom, southwest coast, India, 17 August 2018 (CAH/INV/ISO 0320); 1 ovigerous female (13.8.0 mm), from Muttom, southwest coast, India, 17 August 2018 (CAH/INV/ISO 0321); 1 ovigerous female (partially dissected) (12.5 mm), from Muttom, southwest coast, India, 17 August 2018 (CAH/INV/ISO 0322).

### Description

*Holotype female* (Figs. 1, 2, 3, 4, 5, 6, 7, 8): *Body* sub-oval, asymmetrical, slightly twisted to one side, slightly valuated dorsally, 1.50 times longer than wide, widest at pereonite 3. *Cephalon*, conspicuous, anterior margin slightly triangular, 1.20 times wider than long, slightly constricted anterior to eyes, posterior margin smoothly rounded. *Eyes* ovate, distinct, visible dorsally, with distinct ommatidia, 0.35 times width of cephalon. *Coxae* 2–5 slightly visible dorsally; 6 and 7 moderately visible. *Pereon* medially broad, dorsally convex, twisted to one side. Pereonite 4 longest, 7 shortest. Pereonites gradually increase in width from 1–3, gradually decreasing posteriorly. Posterolateral margins of pereonite 1 not produced; 2–7 progressively produced. Posterolateral margins of pereonite 7 slightly indented. *Pleon* short 15% BL, 0.70 times as wide as maximum pereon width; Pleonite 1 and 2 slightly overlapped by pereonite 7. *Pleonite* 1 shorter and narrower than pleonite 2; pleonites 2–5 progressively narrower towards posterior; lateral borders free, slightly expanded, pleonite 5 longest in dorsal view. *Pleotelson* 1.35 times wider than long, posterior margin hemispherical, anterior margin slightly narrower than pleon.

*Antennula* proximal article slightly expanded; article 8 with few terminal setae. *Antenna* with 12 articles, 1.6 times longer than antennula, article 8 with few on posterodistal angle, article 12 with few terminal setae. Both antennula and antenna with tuft of very fine setae on distal margin of all articles. *Mandible* with prominent molar lobe; *palp* article 3 with 1 long and 8–10 short setae on distolateral margin, article 2 with 2 or 3 setae. Maxillule with 1 large and 3 small slightly recurved apical **RS**. Maxilla basally widest; inner median lobe with 1 and outer lateral lobe with 4 small, slightly recurved **RS**. Maxilliped with marginal plumose setae; article three with 2 terminal recurved **RS**.

*Pereopod 1* basis, 1.70 times as long as greatest width; ischium 0.75 times as long as basis; propodus as long as wide; dactylus slender, as long as propodus, 3 times as long as basal width. *Pereopod 2* basis, 2.33 times as long as greatest width; ischium 0.60 times as long as basis; merus lateral margin with bulbous protrusion; propodus 1.50 times as long as wide; dactylus slender, 0.65 times as long as propodus, 1.65 times as long as basal width. *Pereopod 3* basis, 2.1 times as long as greatest width; ischium 0.70 times as long as basis; propodus 1.4 times as long as wide; dactylus slender, 1.1 times as long as propodus. *Pereopod 4* basis, 2.85 times as long as greatest width; ischium 0.70 times as long as basis; propodus 1.8 times as long as wide; dactylus short, 0.7 times as long as propodus. *Pereopod 5* basis, 2.63 times as long as greatest width; ischium 0.70 times as long as basis; propodus 1.8 times as long as wide; dactylus slender, 0.8 times as long as propodus. *Pereopod 6* basis 2.5 times as long as width; ischium 0.85 times as long as basis; propodus 0.77 times as long as wide, 0.40 times as long as ischium; dactylus as long as propodus, 2.10 times as long as basal width. *Pereopod 7* basis, 2.50 times as long as greatest width; ischium 0.80 times as long as basis; merus 0.65 times as long as wide, 0.33 times as long as ischium; propodus 1.75 times as long as wide, 0.58 times as long as ischium; dactylus 0.85 times as long as propodus, 2.40 times as long as basal width.

*Brood pouch*; oostegites of pereonites 4 and 5 larger than those of pereopods 2 and 3; anteriorly covered by maxilliped oostegial lobes.

Pleopods 1–5 endopods without proximo-medial lobe. *Pleopod 1*, exopod 1.6 times as long as wide, lateral margin slightly convex, distally broadly rounded, mesial margin convex; endopod 0.9 as long as exopod, 1.7 times as long as wide, lateral margin weakly convex, distally broadly rounded; peduncle 3 times as wide as long. *Pleopod* 2 without appendix masculina. *Pleopod 5* exopod 1.35 times as long as wide, lateral margin convex, distally rounded, mesial margin convex; endopod 0.85 times as long as exopod, 1.1 times as long as wide, distally broadly rounded; peduncle 2 times as wide as long.

*Uropod* 0.60 times as long as pleotelson; peduncle 0.5 times as long as exopod, 1.1 times as wide as long, lateral margin without setae; rami without marginal setae, apices narrowly rounded. *Exopod* 2.6 times as long as greatest width, 1.4 times as long as endopod, lateral margin convex. *Endopod* 0.7 times as long as exopod, apically narrowly rounded, exopod curved to mesial, 1.75 times as long as greatest width, mesial margin concave, lateral margin convex.

***Male*** (**Figs.** **9**, **10**, **11**): Body symmetrical, smaller than ovigerous female, 2.00–2.10 times longer than wide. Cephalon anterior border slightly triangular, 1.50–1.60 times wider than long, not immersed in pereonite 1. Eyes more prominent than those of ovigerous female. Pereonites more or less equal in width; pereonite 1 longest. Coxae of anterior pereonites not visible dorsally, coxae 6 and 7 posterior part visible. Pleonite 1 slightly overlapped laterally by pereonite 7. Pleonites subequal in length and width, similar to those of female. Pleotelson 1.50 times wider than long, shorter than pleonite 5, posterior margin broadly rounded.

Antennula, antenna maxillule, and mandible similar to those of ovigerous female. Maxilla basally widest; inner median lobe with 1 and outer lateral lobe with 3 small, slightly recurved **RS**. Maxilliped slightly narrower than in non-ovigerous female, article three with 2 terminal recurved **RS**.

Pereopods similar to those of ovigerous female. Penes conical, apices blunt and medially united. Appendix masculina straight, small, shorter than endopod.

Uropod slightly larger than in female, about half the length of pleotelson; rami unequal in length, curved and apically rounded, exopod longer than endopod.

***Transitional stage*** (Fig. **12**): Body 2.0 times longer than wide; slightly hunched towards one side, cephalon similar to that of male. Pereonites, pleonites, antennula, antenna, and mandible palp similar to those of ovigerous female and maxilla, maxilliped similar to those of male. Coxae similar to that of female. Penes not prominent. Pleotelson 1.40 times wider than long, shorter than pleonite 5. Uropods longer than that of female, reaching up to 0.50 length of pleotelson. Rami unequal, similar to those of male. Pereopods and pleopods similar to those of male and ovigerous female.

***Premanca*** (Fig. **13**): Elongated and transparent body, 2.80–3.00 times longer than wide. Eyes prominent and conspicuous in dorsal view. Cephalon 1.20 times wider than long. Yolk globules are visible in pereon, between pereonites 1 and 6. Pereonite 2 widest; gradually decreasing in width from 4–7. All pleonites visible and subequal in length and width. Pleotelson 1.40 times wider than long, without plumose setae. Antennula with 8 articles, extending slightly beyond anterior margin of pereonite 1. Antenna longer than antennula, with 12 articles; all articles without setae and spinules, extending beyond posterior margin of pereonite 1. Mouthparts not well developed; mandible palp articles without setae and spines; maxillule, maxilla and maxilliped with poorly developed apical spines. Apical spines not recurved. Six pereopods, all pereopods without spines. Propodus and dactylus of pereopods not toothed. Pleopods not visible in dorsal view. All pleopods without plumose setae. Uropod rami subequal, extending beyond distal margin of pleotelson, apically rounded without plumose setae.

***Manca*** (Fig. **14**): Body elongate, transparent, 2.9–3.0 times longer than wide. Eyes distinct, similar to those of pre-manca. Cephalon 1.40 times wider than long. Pereonite 3 widest, progressively decreasing in width from 3–7. Pereonites subequal in length; 7 short and narrow. All pleonites visible, similar to those of pre-manca. Pleotelson slightly wider than long; apical margin with 6–8 plumose setae. Antennula with 8 articles, extending beyond anterior margin of pereonite 1; all articles with few small setae; article 8 with 2 elongate setae. Antenna longer than antennula, with 12 articles extending beyond anterior margin of pereonite 2; all articles with small setae and article 7 with 1 elongate seta; article 12 with few setae and terminal aesthetascs. Article 3 of mandible palp with 2 marginal setae. Maxillule, maxilla, and maxilliped similar to those of male stage. Six pereopods; pereopods 1–3 without robust setae Merus, carpus and propodus of pereopod 3–6 with few spines on distal margin. Pleopods not distinctly visible in dorsal view. Pleopod 1–5 with 6–8 plumose setae on apical margin of both endopod and exopod. Uropod rami endopod broader than exopod, extending strongly beyond distal margin of pleotelson; exopod with 4–6 and endopod with 6–8 plumose setae.

***Variation of adult female*:** *Body* sub-oval, slightly twisted to either left or right side, 1.50–1.80 times longer than wide. *Cephalon*, 1.20–1.30 times wider than long. *Pleotelson* 1.25–1.40 times wider than long. Maxilliped without oostigite lobe in non-ovigerous female. The number of eggs or larvae in brood pouch ranges from 70–220 according to size of female.

***Body Size:*** ovigerous female 9.0–14.0 mm; non-ovigerous female 8.5–13.0; male 7.0–9.0 mm; transitional stage 8.0–11.0 mm; manca 2.2–3.0 mm; premanca 1.8–2.2 mm.

***Colour:*** Female, male and transitional stage of light pink colour with scattered chromatophores throughout pereon; premanca and manca clear with scattered chromatophores.

***Distribution:*** Neendakara, Quilon, Kerala coast, (Kumar & Bruce, 1997; Aneesh et al., 2020a; present study), Muttom, Tamil Nadu, southwest coast, India (Aneesh et al., 2020a; present study).

***Host:*** Known only from the type host, *Samaris cristatus* (Kumar & Bruce, 1997; Aneesh et al., 2020a; present study).

***Etymology:*** The specific name *‘tiranga’*, is derived from two Hindi words, *Tin* and *Ranga* (originally from the Sanskrit word *Tri* and *Ranga*) which colloquially means the tri-colour of India's flag. Furthermore, ‘*Tiranga Point’* is a location on the Moon near the lunar south pole where Chandrayaan-2's lander Vikram crashed. The site was named on 26 August 2023. It is located at the coordinates 70.8810° S 22.7840° E and it lies between ‘Manzinus C’ and ‘Simpelius N’ craters.

**Remarks:*** Sandythoa tiranga* **sp. nov.** can be distinguished from all other congeners by: Body widest at pereonite 3; pleon 0.6-0.7 as wide as widest pereomere; antenna with 12 articles; Pleonite 2 widest; pleonites 2–5 progressively narrower towards posterior; pleotelson 1.35 times wider than long, posterior margin hemispherical; uropod exopod 1.4 times as long as endopod. Interspecific character differences between the species of *Sandythoa* **gen. nov.** are listed in Table 1.

**Table 1:** Interspecific morphological characters of *Sandythoa tiranga* **sp. nov.,** *Sandythoa arnoglossi* (Trilles & Justine 2006) **comb. nov.** *Sandythoa parabothi* Trilles & Justine, 2004) **comb. nov.** *Sandythoa samariscii* (Shiino, 1951) **comb. nov.**, and *Sandythoa moritakii* (Saito & Yamauchi, 2016) **comb. nov.** collected from the original description/redescriptions (see Shiino, 1951; Trilles & Justine, 2004, 2006; Saito & Yamauchi, 2016).

Characters	*S. tiranga* **sp. nov.**	*S. arnoglossi* **comb. nov.**	*S. parabothi* **comb. nov.**	*S. samariscii* **comb. nov.**	*S. moritakii* **comb. nov.**
Body shape/proportions	1.5 as long as greatest width	1.5 as long as greatest width	1.4 as long as greatest width	1.8 as long as greatest width	2.2 as long as greatest width
Number of articles in antenna	12	18	18	10	11
Widest pereomere	3	4	4	5	4
Pleon maximum width to widest pereomere	0.6–0.7	0.53	0.45	0.52	0.7
Pleonite width	2 widest; pleonites 2–5 progressively narrower towards posterior	2 widest; 2–5 progressively narrower towards posterior	2 widest; 2–4 progressively narrower towards posterior	3 widest; 3–5 progressively narrower towards posterior	4 widest; 2–5 subequal
Pleotelson	1.35 times wider than long, posterior margin hemispherical	1.6 times as wide as long, hemispherical	1.85 times as wide as long	1.5 times as wide as long.	1.6 times as wide as long, pleotelson semicircular
Uropod	exopod 1.4 times as long as endopod	endopod 1.2 times as long as exopod	endopod 1.1 times as long as exopod	exopod 1.25 times as long as endopod	exopod 1.3 times as long as endopod

***Sandythoa arnoglossi* (**Trilles & Justine, 2006**) comb. nov.**

*Elthusa arnoglossi.*—Trilles & Justine, 2006: 59–66, figs 1–4.

**Remarks: ***Sandythoa arnoglossi* (Trilles & Justine, 2006) **comb. nov.** was described from the bothid fish *Arnoglossus* sp., collected from the Chesterfield Islands, New Caledonia (southwestern Pacific)*.* We place this species in combination with *Sandythoa* **gen. nov.** based on: pleonite 1 is narrow, not more laterally extended than others; body vaulted dorsally, widest at pereonite 3; cephalon not deeply immersed in pereonite 1, anterior margin with acute rostral point, rostrum narrowly rounded, not folded; pereonites 2–7 coxae visible in dorsal view; pleon 0.50 times as wide as maximum pereon width; antennula separated by rostrum, slender, with 8 articles, shorter than antenna; buccal cone obscuring antennal bases; pereopodal dactyli relatively short and strongly curved. Interspecific character differences between the species of *Sandythoa* **gen. nov**. are listed in Table 1.

***Body Size:*** Ovigerous female 9–13 mm; non-ovigerous female 10.5–13 mm; male 6.5–8.5 mm; transitional stage 8.5–9.0 mm (Trilles & Justine, 2006).

***Distribution:*** Chesterfield Islands, New Caledonia (southwestern Pacific) (Trilles & Justine, 2006).

***Host:**** Arnoglossus* sp. (Pleuronectiformes, Bothidae) (Trilles & Justine, 2006).

***Sandythoa moritakii* (**Saito & Yamauchi, 2016**) comb. nov.**

(Figs. 15, 16, 17)

**Figure 15:**
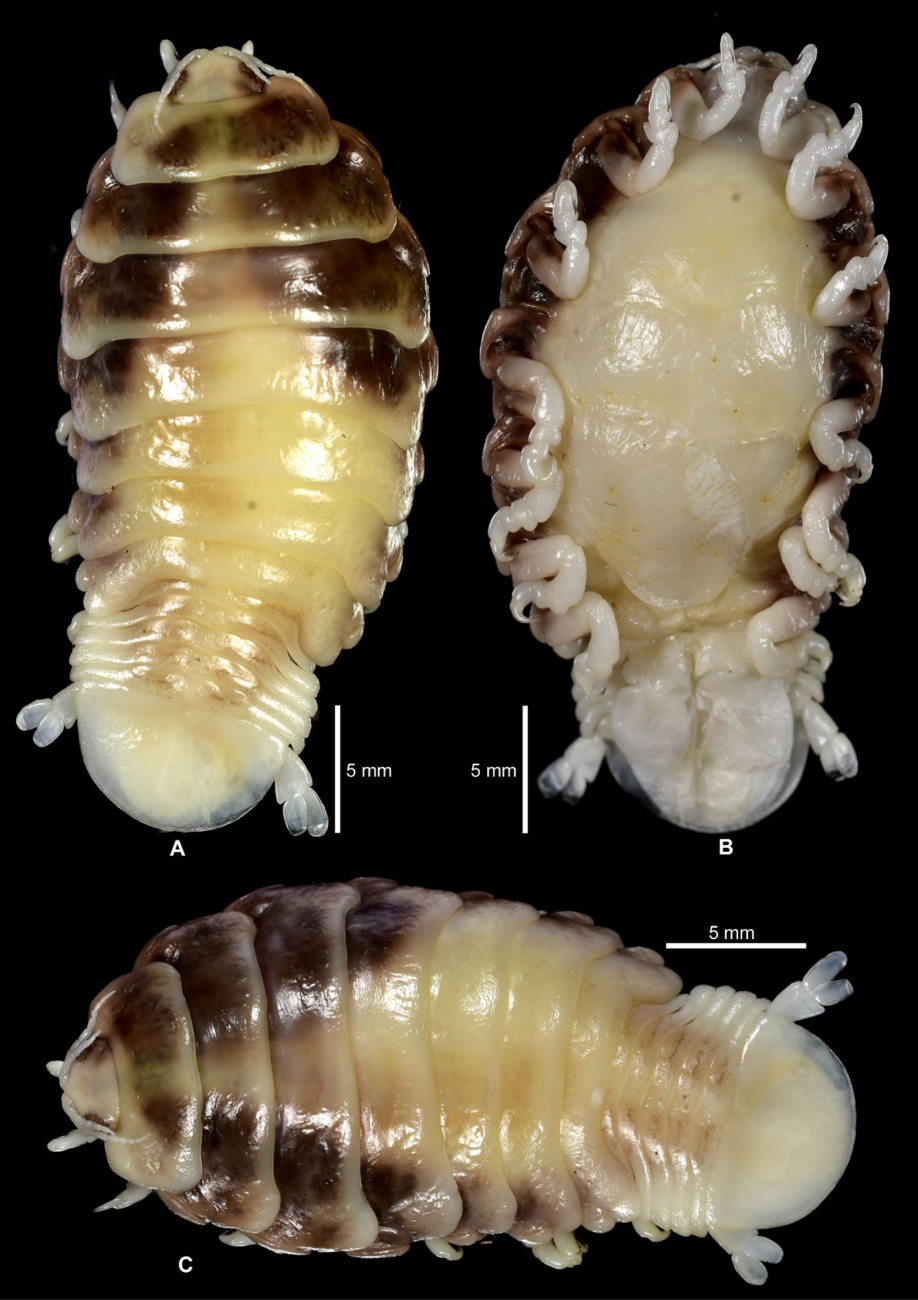
*Sandythoa moritakii* (Saito & Yamauchi, 2016) **comb. nov.** ovigerous female. A, dorsal view; B, ventral view; C, dorso-lateral view.

**Figure 16:**
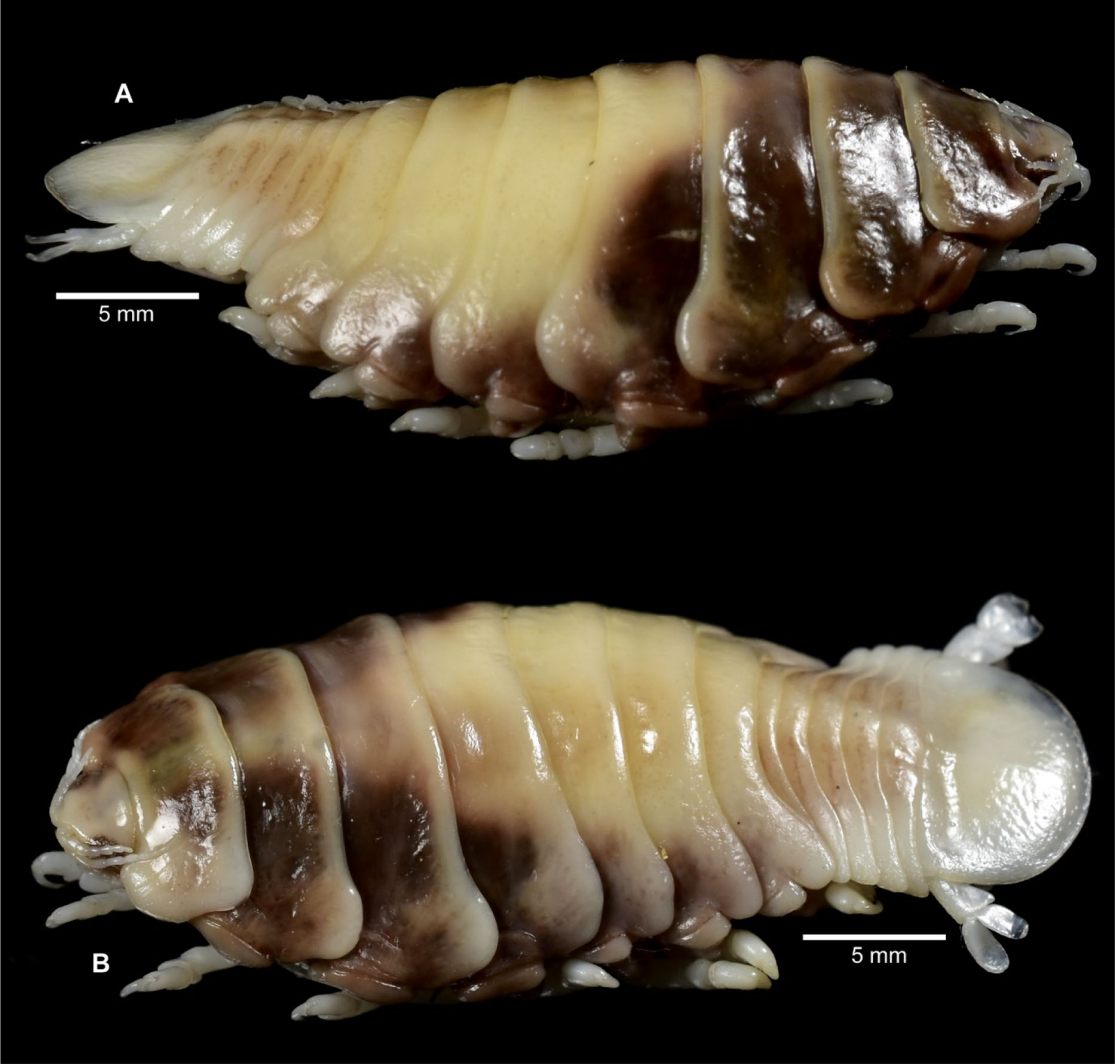
*Sandythoa moritakii* (Saito & Yamauchi, 2016) **comb. nov.** ovigerous female. A-B, lateral views.

**Figure 17:**
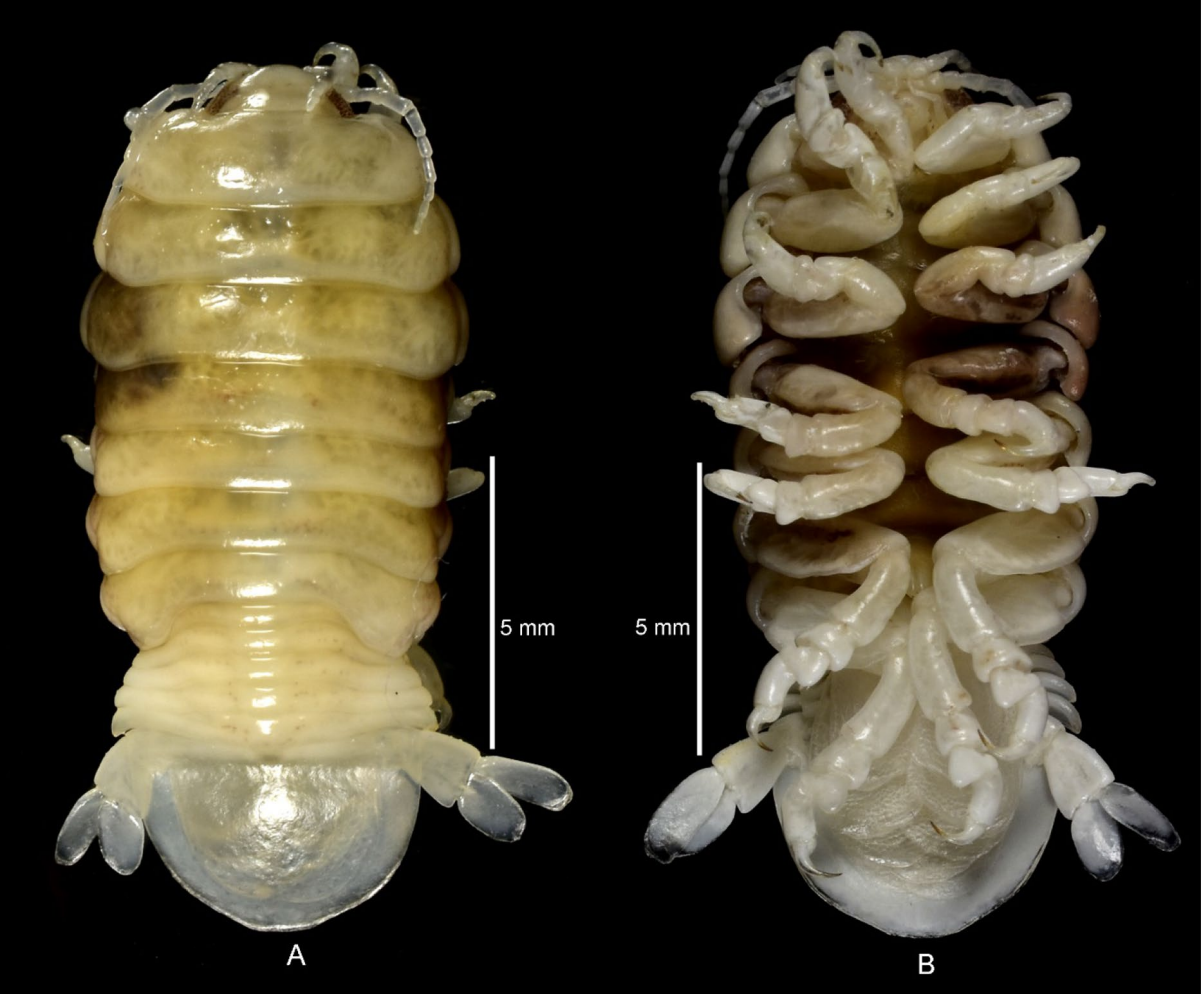
*Sandythoa moritakii* (Saito & Yamauchi, 2016) **comb. nov.,** male. A, dorsal view; B, ventral view.

*Elthusa moritakii*.—Saito & Yamauchi, 2016: 60–66, figs 1–5.

*Materials examined:* 1 ovigerous female (29.5 mm) and 2 ales (9.0 mm; 12.0 mm) from *Ereunias grallator* Jordan & Snyder, off Suruga Bay, Japan.

**Remarks: ***Sandythoa moritakii* (Saito & Yamauchi, 2016) **comb. nov.,** was described from Japan (North Pacific Ocean and East China Sea) based on several specimens collected from the deepwater bullhead sculpin *Ereunias grallator* Jordan & Snyder. Based on the following characters we place the species in combination with *Sandythoa* **gen. nov**.: pleonite 1 is narrower than others; body widest at pereonite 3; cephalon not deeply immersed in pereonite 1, anterior margin with acute rostral point; pereonites 2–7 coxae visible in dorsal view; pleon 0.70 times as wide as maximum pereon width; antennula separated by rostrum, slender, with 8 articles, shorter than antenna; buccal cone obscuring antennal bases; pereopodal dactyli relatively short, strongly curved Interspecific character difference between the species of *Sandythoa* gen. nov. are listed in Table 1.

***Body Size:*** Female 29.5–31.6 mm; male 7.7–18.0 mm.

***Distribution:*** Japan (North Pacific Ocean and East China Sea) (Saito & Yamauchi, 2016; present study)

***Host:*** Known only from the type host *Ereunias grallator* Jordan and Snyder (Saito & Yamauchi, 2016; present study).

## ***Sandythoa parabothi*** (Trilles & Justine, 2004) comb. nov.

*Elthusa parabothi*—Trilles & Justine, 2004: 213–216, figs 1–5.

**Remarks: ***Sandythoa parabothi* (Trilles & Justine, 2004) **comb. nov.,** was originally described from New Caledonia, based on specimens collected from the branchial cavity of lefteye flounders, *Parabothus kiensis* (Tanaka) (Bothidae) collected at depths of 385 to 401 meter (Trilles & Justine, 2004). Based on the following characters we place the species in combination with *Sandythoa* **gen. nov**.: body widest at pereonites 3 and 4; pleonite 1 is narrower than others; cephalon not deeply immersed in pereonite 1, anterior margin with acute rostral point; pereonites 2–7 coxae visible in dorsal view; pleon 0.40 times as wide as maximum pereon width; antennula separated by rostrum, shorter than antenna; pereopods dactyli relatively short, strongly curved. Interspecific character difference between the species of *Sandythoa* **gen. nov**. are listed in Table 1.

***Body Size:*** Female 14.5 mm; male 13 mm.

***Distribution:*** Known only from the type locality New Caledonia (Trilles & Justine, 2004).

***Host:*** Known only from the type host *Parabothus kiensis* (Tanaka) (Bothidae) (Trilles & Justine, 2004).

## ***Sandythoa samariscii*** (Shiino, 1951) comb. nov.

(Fig. 18)

**Figure 18:**
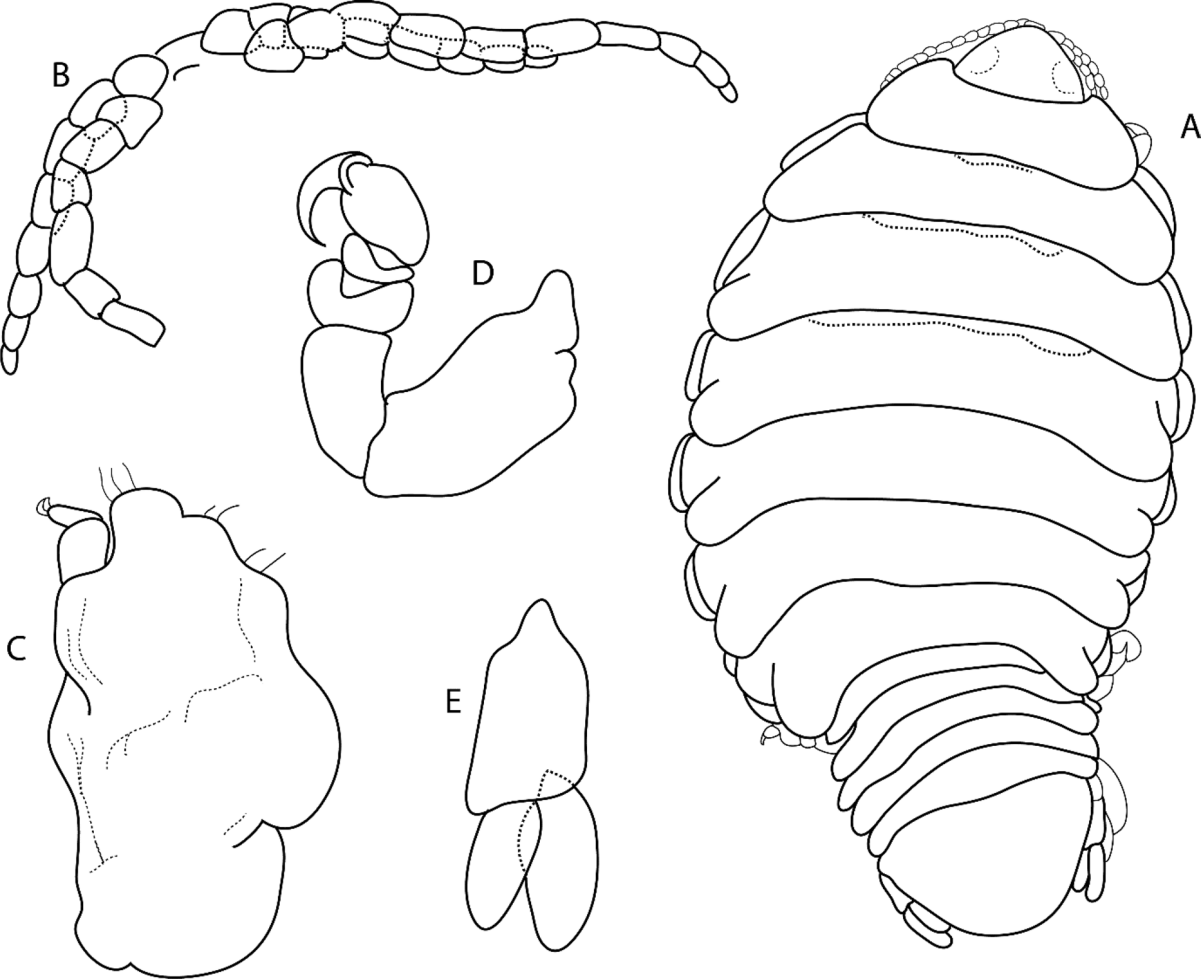
*Sandythoa samariscii* (Shiino, 1951) **comb. nov.** female from *Samariscus japonicus* Kamohara (Samaridae) from Japan. A, dorsal view; B, antennula and antenna; C, maxilliped; D, pereopod 1; E, uropod. (Redrawn from fig. 5 of Shiino (1951)).

*Livoneca samariscii*—Shiino, 1951: 86–87, fig. 5.

*Elthusa samariscii*— Bruce, 1990: 287.

**Remarks: ***Sandythoa samariscii* (Shiino, 1951) **comb. nov.**, was described from the samarid fish *Samariscus japonicus* Kamohara, 1936 from Kochi, Japan. Shiino (1951) minimally described the species and illustrated the dorsal view, buccal cone with antennula and antenna, pereopod 1, maxilliped, and uropod, based on a single ovigerous female; after the original description, it has not been recorded elsewhere. Based on the following characters we place the species in combination with *Sandythoa* **gen. nov**.: dorsally vaulted body, cephalon not deeply immersed in pereonite 1, anterior margin with acute rostral point; rostrum narrowly rounded, not folded, pereonites 2–7 coxae visible in dorsal view, pereonites 7 posterolateral margins partially concealing pleonite 1 and 2; pleon less than 0.70 times as wide as maximum pereon width, pleonite 1 is laterally reduced, pereopod 7 dactyli relatively short, strongly curved. Interspecific character difference between the species of *Sandythoa* **gen. nov.** are listed in Table 1.

***Body Size:*** Female 10.1 mm.

***Distribution:*** Known only from the type locality Kochi, Japan (Shiino, 1951).

***Host:*** Only from the type host, *Samariscus japonicus* Kamohara (Shiino, 1951).

## Discussion

*Sandythoa* **gen. nov.** is the fourth genus of fish parasitic cymothoid originally described from Indian waters. The slightly asymmetrical, but not distorted, body shape permits *Sandythoa* **gen. nov.** to be distinguished from the branchial cymothoid genera *Agarna* Schioedte & Meinert, 1884, *Cterissa* Schioedte & Meinert, 1884, *Kuna* Bunkley-Williams, 1986 and *Ryukyua* Williams & Williams, 1994, all of which have strongly distorted asymmetric body shapes. The simple pleopods, brood pouch without posterior pockets, slender antennae, and pereopodal morphology places the new genus close to genera such as *Brucethoa* Aneesh, Hadfield, Smit, & Kumar, 2020, *Elthusa*, *Glyptothoa, Ichthyoxenos* Herklots, 1870 (marine species only), *Mothocya* A. Costa *in* Hope, 1851 and *Catoessa* Schioedte & Meinert, 1884 (Table 2) (Aneesh et al., 2020b, Helna et al., 2023).

**Table 2:**
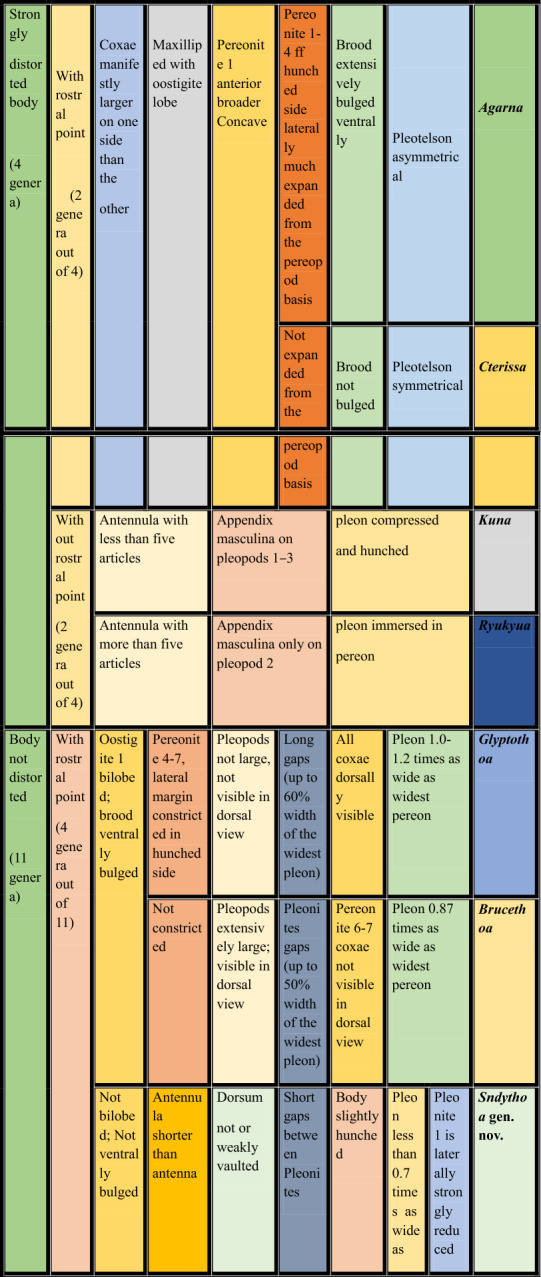

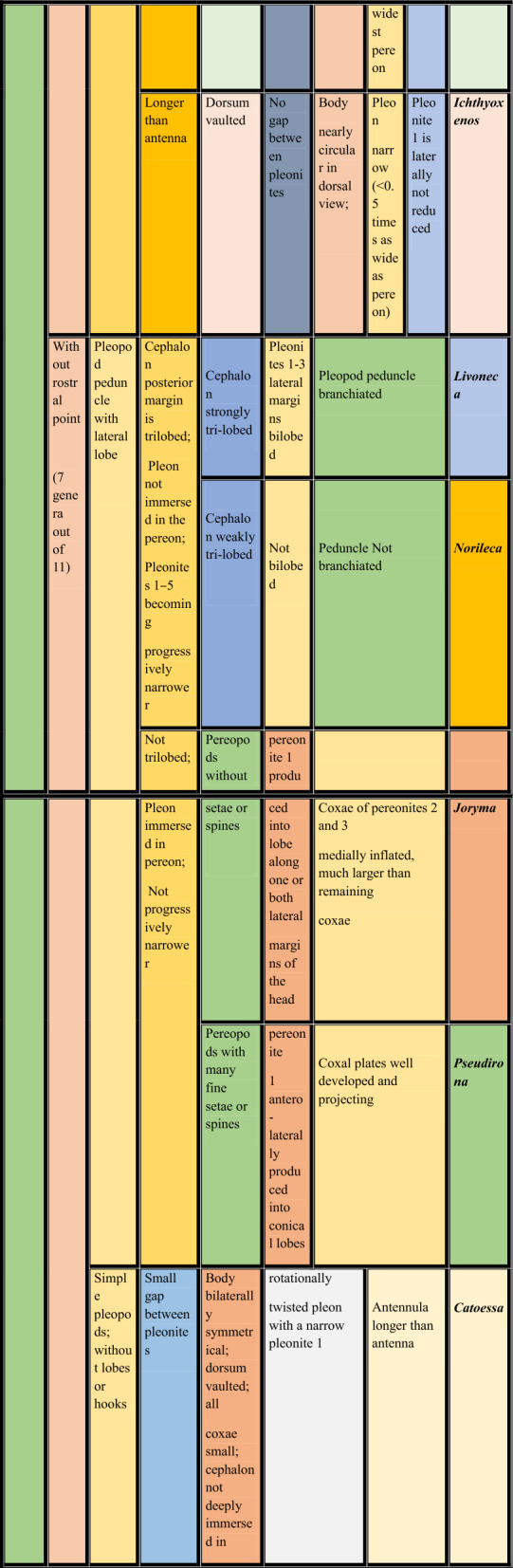

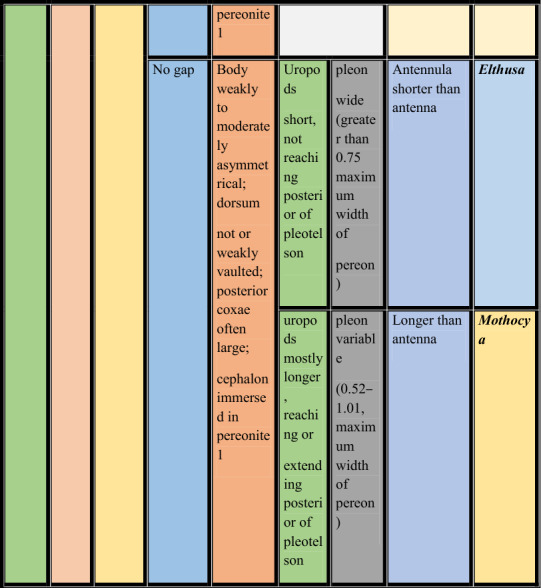
Inter-generic characters of 15 branchial cymothoid genera.

*Sandythoa* **gen. nov.** has an acute ventrally directed rostrum and simple pleopods (without fleshy lobes), which distinguishes it from *Joryma* Bowman & Tareen, 1983*, Livoneca* Leach, 1818*, Norileca* Bruce, 1990*,* and *Pseudirona* Pillai, 1964, all of which have a cephalon without rostrum and pleopods with fleshy lobes (Bruce, 1990; Aneesh et al., 2019, 2020b, Helna et al., 2023).

*Sandythoa* **gen. nov.** can be separated from *Brucethoa* by: pleopods not large, not visible in dorsal view (vs extensively large pleopods, visible in dorsal view in *Brucethoa*) and all coxae visible in dorsal view in (vs the coxae of pereonites 6 and 7 not visible in dorsal view in *Brucethoa*) (Aneesh et al., 2020b, 2024).

*Sandythoa* **gen. nov.** can be separated from *Glyptothoa* by: pleonites lateral margins not constricted (vs pleonites 4–7 lateral margins constricted on hunched side in *Glyptothoa*); pleon less than 0.7 times as wide as widest pereon (vs pleon 1.0–1.2 times as wide as pereon in *Glyptothoa*). Further, oostigite 1 is not bilobed in *Sandythoa* **gen. nov.** (vs oostigite 1 bilobed in both *Glyptothoa* and *Brucethoa*) (Aneesh et al., 2020b, 2024; Helna et al., 2023).

*Sandythoa* **gen. nov.** differs from *Elthusa*, as defined by Aneesh et al., (2020b), in the following characters: cephalon anterior margin with acute ventrally directed rostrum in *Sandythoa* **gen. nov.** (vs dorsally truncate in *Elthusa*); buccal “cone” anteriorly positioned, overriding antennal bases in *Sandythoa* **gen. nov.** (vs not anteriorly positioned, not overriding antennal bases in *Elthusa*); pleonites 1–5 with free lateral margins *Sandythoa* **gen. nov.** (vs pleonites 2–5 or 3–5 in *Elthusa*); short gaps are present between all pleonites *Sandythoa* **gen. nov.** (vs without gaps in *Elthusa*) (see Aneesh et al., 2020b; Helna et al., 2023).

The genus *Catoessa* does have small gaps between the pleonites but differs from *Sandythoa* **gen. nov.** in having: a rotationally twisted pleon with a narrow pleonite 1, the anterior margin of the cephalon lacking a rostral point, and the uropods extend about halfway along to beyond the posterior margin of the pleotelson (Bowman & Tareen, 1983; Bruce, 1990; Trilles et al., 2012; Aneesh et al., 2020a; Helna et al., 2023).

*Mothocya* differs from *Sandythoa* **gen. nov.** in having the antennula being both distinctly longer and stouter than the antenna, lacking a distinct rostral point, the uropods extending to or beyond the posterior margin of the pleotelson, and the absence of a gap between pleonites (see Bruce, 1986; Hadfield et al., 2015; Aneesh et al., 2016; 2020b; Kawanishi et al., 2023; Helna et al., 2023).

The genus *Ichthyoxenos* includes both flesh burrowers as well as gill-attaching species inhabiting both freshwater and marine water (Bruce, 1990). *Sandythoa* **gen. nov.** differs from *Ichthyoxenos* by following features: body slightly hunched (vs body strongly ovate and nearly circular in dorsal view in *Ichthyoxenos*); short gaps are present between all pleonites (vs no gap between pleonites in *Ichthyoxenos*); pleonite 1 laterally reduced (vs pleonite 1 laterally not reduced in *Ichthyoxenos*).

## Conclusions

The branchial attaching species described here was found to differ consistently from all other known cymothoid genera; we describe *Sandythoa* **gen. nov.** with the type species *S. tiranga* **sp. nov.** The new genus *Sandythoa* is the 44th genus in the family and the fourth genus originally described from India. Based on the generic characters, four other species of *Elthusa* are now transferred into the new genus: *S. arnoglossi* (Trilles & Justine, 2006) **comb. nov.**; *S. parabothi* (Trilles & Justine, 2004) **comb. nov.**; *S. samariscii* (Shiino, 1951) **comb. nov.**; and *S. moritakii* (Saito & Yamauchi, 2016) **comb. nov.**

## Data Availability

Type and voucher specimens were deposited in the collections of Western Ghats Field Research Centre of Zoological Survey of India, Kozhikode (ZSI/WGRC).
